# SLC22A3 regulates ferroptosis in the mesenchymal subtype of triple-negative breast cancer by modulating histone H3K4 serotonylation

**DOI:** 10.1038/s41419-026-09057-6

**Published:** 2026-07-09

**Authors:** Dongsheng Zhai, Wang Li, Guo Chen, Wenli Zhang, Yue Hu, Lele Deng, Dengguo Li, Han Cheng, Jixiang Zhou, Mingkai Li, Li Wang, Lu Zifan

**Affiliations:** 1 Department of Pharmacology, School of Pharmacy, Fourth Military Medical University, Xi’an, Shaan xi China; 2 State Key Laboratory of Holistic Integrative Management of Gastrointestinal Cancers, Biotechnology Center, School of Pharmacy, Fourth Military Medical University, Xi’an, China; 3 Translational medicine Center of Shaanxi Provincial People’s Hospital, Xi’an, Shaanxi China; 4 Department of Experimental Surgery, Tangdu Hospital, Fourth Military Medical University, Xi’an, Shaanxi China; 5 School of Aerospace Medicine, Fourth Military Medical University, Xi’an, China

**Keywords:** Breast cancer, Cancer therapeutic resistance, Mechanisms of disease, Cancer therapy

## Abstract

Triple-negative breast cancer (TNBC) remains a challenging and clinically aggressive subtype due to its heterogeneity and high mortality rate. Recent molecular subtyping has identified distinct TNBC subgroups with varying therapeutic responses, highlighting the need for targeted therapeutic strategies. The mesenchymal (MES) subtype is characterized by low immune cell infiltration, cancer stem cell-like features, and resistance to multiple drugs. Ferroptosis, a form of iron-dependent cell death, has emerged as a promising therapeutic strategy in TNBC due to the abundance of iron and lipids in tumor cells. However, ferroptosis sensitivity varies across different TNBC subtypes. Notably, the MES subtype exhibits resistance to ferroptosis despite elevated iron levels, due to impaired ferroptosis-executing mechanisms. This study investigates the role of SLC22A3, an organic cation transporter, which is enriched in MES tumors and positively correlates with markers of tumor stem cells. High SLC22A3 expression in MES-TNBC cells modulates serotonin uptake and metabolism, conferring ferroptosis resistance through two pathways. First, 5-HT(serotonin) acts as a radical-trapping antioxidant, eliminating lipid peroxides and inhibiting ferroptosis. Second, 5-HT induces histone serotonylation, which enhances histone methylation and facilitates the recognition of methylated histones by transcriptional initiation factors. This process activates SIRT1 transcription, inhibiting MAOA transcription mediated by FOXO1, thereby reducing 5-HT degradation and promoting ferroptosis resistance. Moreover, we identified potential SLC22A3 inhibitors and their synergistic combinations with ferroptosis inducers or cisplatin, which suppress tumor growth in both MES-subtype TNBC patient-derived organoids and in vivo, offering a promising strategy for personalized therapy. These findings suggest that targeting SLC22A3, along with ferroptosis inducers, may offer a promising therapeutic strategy for patients with MES-subtype TNBC.

**Figure:**
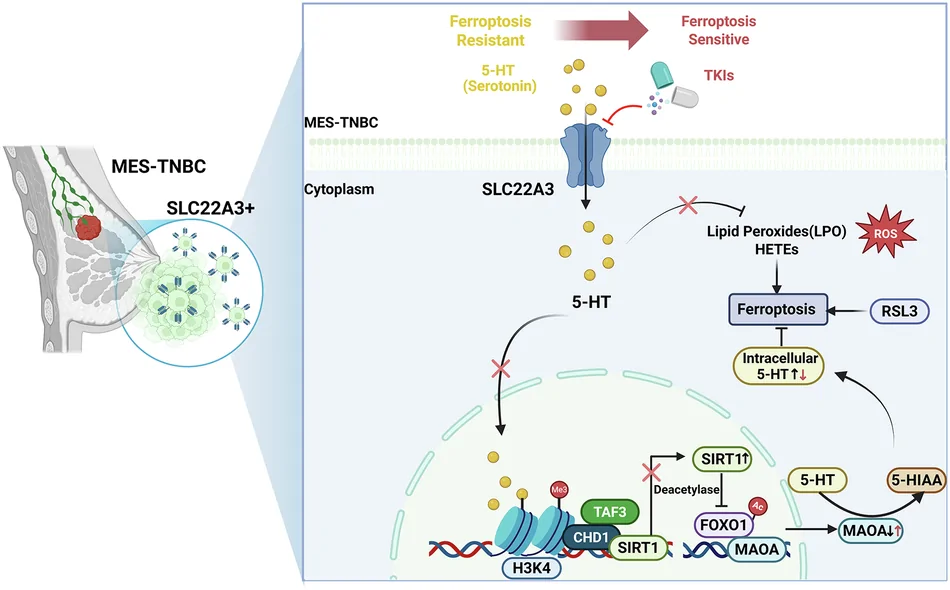
SLC22A3 affects serotonin uptake and metabolism in MES-TNBC cells, providing ferroptosis resistance through two mechanisms. 5-HT acts as a radical-trapping antioxidant, eliminating lipid peroxides and inhibiting ferroptosis. In addition, 5-HT induces histone serotonylation, which enhances histone methylation and facilitates the recognition of methylated histones by transcription initiation factors. This process activates SIRT1 transcription, inhibiting MAOA transcription mediated by FOXO1, thereby reducing 5-HT degradation and promoting ferroptosis resistance. TKIs served as SLC22A3 inhibitors and their combinations with RSL3 or cisplatin, which inhibit tumor growth in vivo, offering new personalized treatment options for MES-TNBC patients.

SLC22A3 affects serotonin uptake and metabolism in MES-TNBC cells, providing ferroptosis resistance through two mechanisms. 5-HT acts as a radical-trapping antioxidant, eliminating lipid peroxides and inhibiting ferroptosis. In addition, 5-HT induces histone serotonylation, which enhances histone methylation and facilitates the recognition of methylated histones by transcription initiation factors. This process activates SIRT1 transcription, inhibiting MAOA transcription mediated by FOXO1, thereby reducing 5-HT degradation and promoting ferroptosis resistance. TKIs served as SLC22A3 inhibitors and their combinations with RSL3 or cisplatin, which inhibit tumor growth in vivo, offering new personalized treatment options for MES-TNBC patients.

## Introduction

Triple-negative breast cancer (TNBC) is defined by the absence of estrogen receptor (ER), progesterone receptor (PR), and human epidermal growth factor receptor 2 (HER2) expression [1, 2]. Due to the heterogeneity of the disease, it remains the most difficult breast cancer subtype to treat clinically and is associated with a very high mortality rate [2, 3]. Thus, the discovery of novel actionable molecular targets is urgent. In recent years, molecular subtyping efforts based on gene expression profiling, immunophenotyping, and metabolic characteristics have identified multiple TNBC subgroups with distinct molecular profiles and differential therapeutic responses. Lehmann et al. defined four subtypes based on gene expression profiles: basal-like 1 (BL1), basal-like 2 (BL2), mesenchymal (M), and luminal androgen receptor (LAR) [3, 4]. Similarly, Burstein et al. proposed a four-subtype classification system based on the genetic profiles of 198 TNBC tumors: basal-like immunosuppressed (BLIS), basal-like immune-activated (BLIA), mesenchymal (MES), and luminal androgen receptor (LAR) [5]. Furthermore, Shao et al. conducted a comprehensive analysis integrating genomic and transcriptomic data of TNBC tumors, along with data on driver gene types and mutations. They classified TNBC into four transcriptome-based subtypes: luminal androgen receptor (LAR), immunomodulatory (IM), basal-like immune-suppressed (BLIS), and mesenchymal-like (MES) [6]. Biomarkers capable of distinguishing between these molecular subtypes were also identified. Specifically, the MES subtype exhibits an immunohistochemical staining profile of AR⁻, CD8⁻, FOXC1⁻, DCLK1⁺ [7]. The MES subtype accounts for ~15–20% of TNBCs and exhibits markedly distinct characteristics compared to other subtypes. Its primary features include low levels of immune cell infiltration, cancer stem cell (CSC)-like features (characterized by JAK/STAT3 signaling pathway activation), and genomic hypermethylation [6]. Furthermore, MES-TNBC demonstrates resistance to multiple therapeutic agents [4].

Ferroptosis is a form of programmed cell death that has been extensively studied in recent years. The accumulation of intracellular free iron (particularly ferrous iron, Fe²⁺ is a key initiating factor in the occurrence of ferroptosis) [8, 9]. Iron generates a large amount of reactive oxygen species (ROS) through the Fenton reaction, which then attacks polyunsaturated fatty acids (such as arachidonic acid and linoleic acid) on the cell membrane, directly inducing lipid peroxidation [10–12]. This ultimately disrupts the integrity of the cell membrane, leading to cell death. Due to plentiful iron and lipids in TNBC, inducing ferroptosis represents a potential therapeutic strategy [13, 14]. A variety of studies have reported that ferroptosis inducers such as RSL3 and erastin induce cell death in breast cancer cells. Recently, Shao et al. have revealed that the ferroptosis phenotypes of four TNBC molecular subtypes demonstrate substantial heterogeneity in both transcriptomics and metabolomics by utilizing integrated multi-omics data from a large TNBC cohort [15]. They found that LAR subtype was hypersensitive to ferroptosis inducers owing to the upregulation of oxidized phosphatidylethanolamines and glutathione metabolism (especially GPX4) [15]. However, the MES subtype exhibited a state of disrupted iron metabolism, characterized by dysfunctional iron-related pathways, diminished activity in fatty acid (FA) metabolism and ROS pathways, as well as lower abundance of glutathione peroxidase 4 (GPX4) and arachidonic acid 15-lipoxygenase (ALOX15) expression compared to LAR-type breast cancer cells [15]. Paradoxically, although MES tumors show enhanced iron accumulation via upregulated uptake pathways, their impaired ferroptosis-executing machinery (GPX4/ALOX15 deficiency) and compromised FA/ROS metabolism collectively confer ferroptosis resistance.

The *SLC22A3* gene encodes organic cation transporter belonging to the SLC22A family (SLC22A1-3 or OCT1-3), which facilitates the transmembrane transport of a range of exogenous and endogenous organic cations, including norepinephrine, dopamine, histamine, and certain drugs [16, 17]. It is crucial for drug transport and cellular detoxification. It has been reported that SLC22A3 is closely associated with the prognosis and drug resistance of various tumors [16, 18–20]; however, its role varies across different types of tumors. Studies have reported that overexpression of SLC22A3 is significantly associated with prolonged survival in patients with pancreatic cancer and glioblastoma multiforme [21]. On the other hand, some scholars have found that high expression of SLC22A3 predicts poor prognosis in patients with lung squamous cell carcinoma, colorectal cancer, and cervical cancer [16, 18, 20]. However, the role of SLC22A3 in breast cancer, particularly in the MES subtype of triple-negative breast cancer, has not been addressed elsewhere.

Based on multi-center sample molecular subtyping analysis, this current work revealed that in the MES subtype of triple-negative breast cancer, SLC22A3 was enriched in synaptic membrane-associated regions. High expression of SLC22A3 showed a significant positive correlation with the expression of 5-HT, the JAK2-STAT3 signaling pathway (a marker of MES tumor stem cells), and FOXP3 (an immunosuppressive marker molecule). Depletion of SLC22A3 significantly enhanced the sensitivity of MES subtype cells to ferroptosis. Mechanistic exploration revealed that SLC22A3 deletion significantly reduced H3K4me3 and histone serotonylation levels, decreased SIRT1 transcriptional activity, and inhibited SIRT1-mediated deacetylation regulation of FOXO1. This ultimately promoted MAOA-mediated degradation of 5-HT and exacerbated oxidative stress in tumor cells, leading to increasing sensitivity to ferroptosis. Furthermore, we discovered that four Tyrosine Kinase Inhibitors (TKI) drugs (Brigatinib, Ceritinib, Pacritinib, and Crizotinib) could significantly inhibit SLC22A3 activity, reduce intracellular 5-HT levels, and enhance the sensitivity of TNBC cells to ferroptosis. Moreover, the combined administration of Pacritinib with ferroptosis inducer RSL3 significantly suppressed tumor growth. Collectively, our findings indicated that SLC22A3 might serve as a novel potential target for personalized therapy in the MES subtype of triple-negative breast cancer.

## Methods

### Antibodies and reagents

Detailed information on the antibodies and regents used is provided in Supplementary Data 2.

### Cell culture and reagents

Human breast cancer cell lines (HCC1806, BT-549, MDA-MB-231, MDA-MB-453, HCC1937, MCF-7) were purchased from ATCC All cell lines were authenticated. All cell lines had been authenticated in 2021 by STR profiling using the AmpFlSTR™. Through short tandem repeat profiling and were used for less than six months within 15–20 passages. All cell lines were cultured in the ATCC-recommended medium containing 10% FBS and 1% streptomycin/ penicillin at 37 °C with 5% CO_2_.

### Extrapolating the clustering results to TCGA TNBCs

We extrapolated our mRNA-based subtypes to the TCGA TNBCs using the R package (https://cran.r-project.org). The TNBC samples were collected from TCGA, which were reported as the previous studies (Supplementary Data 1). The expression data from our study and the log2(RSEM + 1) values of TCGA TNBCs were normalized within each data set (using the “scale” function in R with default parameters).

### Clinical breast cancer samples

Human breast cancer and paired adjacent normal tissues (at least 5 cm away from the tumor), primary and paired metastatic tumor tissues were collected from patients with breast cancer without previous radiotherapy or chemotherapy at the TangDu Hospital of Air Fourth Military Medical University (Clinical Trial Number: GKJ-Y-202303-189). Written informed consent was obtained from all the patients. All procedures were conducted in accordance with the Declaration of Helsinki and approved by the Ethics Committee of Air Fourth Military Medical University (no. 20140603-1).

The tumor subtypes in this study were confirmed as the previous studies described [7]. The immunohistochemical makers classified TNBCs into five subtypes based on the staining results: (a) IHC-based luminal androgen receptor (IHC-LAR; AR-positive(+), (b) IHC-based immunomodulatory (IHC-IM; AR-negative(-), CD8(+)), (c) IHC-based basal-like immune-suppressed (IHC-BLIS; AR(-), CD8(-), FOXC1(+)), (d) IHC-based mesenchymal (IHC-MES; AR(-), CD8(-), FOXC1(-), DCLK1(+)), and (e) IHC-based unclassifiable (AR(-), CD8(-), FOXC1(-), DCLK1(-)). The colllected samples were further classified using the Wenfuxi diagnostic kit (ShuWen biology) developed by Fudan University. The subtypes of tumor samples were showed in Supplementary Data 2.

### Single-cell sequencing data analysis

For single-cell metabolic activity analysis, scRNA-seq datasets from TCGA (GSE176078) were integrated with publicly available scRNA-seq datasets using the Seurat R package (v4.0.3). Subsequently, unsupervised clustering analysis, dimensionality reduction, and cell type annotation were carried out according to established protocols. Cell type assignment was based on known gene markers of various cell types.

### Plasmids, small-interfering RNA, and lentivirus-mediated RNA interference

shRNAs against SLC22A3, MAOA and SIRT1 and the control (shNC), were cloned into the GV493 vector (GeneChem). The cells were infected with a lentiviral vector (GeneChem) expressing sh-SLC22A3, MAOA and SIRT1 or shNC for 12 h. The infected cells were cultured in a medium containing puromycin for 2 weeks to establish the engineered cells. The detailed sequences are provided in Supplementary Data 2.

### IHC staining and score

The tissue slides were washed with PBS after deparaffinization and hydration and then boiled in citrate buffer at 100 °C for 15 min. After blocking endogenous peroxidase, slides were incubated at 4 °C overnight with JAK1, SLC22A3, STAT3, FOXP3 and 5-HT antibodies. After washing with PBS, the slides were incubated with a secondary antibody for 30 min at room temperature. Sections were stained with DAB and counterstained with hematoxylin staining solution according to the manufacturer’s instructions. The strength of IHC staining was calculated by image J. Five views of each slide were quantified by software.

### Immunofluorescence analysis

The slides were washed with PBS, fixed with 4% formaldehyde for 20 min, and permeabilized with 0.5% Triton X-100 for 30 min. The cells were blocked with 5% goat serum for 1 h at 4 °C and incubated SLC22A3, ICAM and 5-HT antibody for 30 min. The cells were washed and incubated with the DyLight 594 conjugated goat anti-rabbit IgG for 30 min, and the cell nuclei were stained with DAPI. The cells were finally observed; their images were captured by microscopy (Olympus IX71) and processed by the Cell Sens imaging software.

### Cell viability assay

We assessed cell viability using the CCK8 assay according to the manufacturer’s instructions. Cells were seeded at a density of 10^4^ cells per well in 24-well plates containing 500 µL of DMEM. Following the specified treatment, CCK8 reagents from Beyotime Biotechnology were introduced to the culture medium and incubated for 2 h. Subsequently, the optical densities of the wells were measured at 450 nm using a microplate reader. The percentage of cell viability was determined by comparing the experimental cells to normal cells.

### Co-immunoprecipitation and western blot analysis

Tumor tissues and cells (BT-549 and CAL-120) were lysed in RIPA lysis buffer containing a protease and phosphatase inhibitor cocktail. Then, the lysates were incubated with the indicated antibodies for 12 hours at 4 °C and mixed with protein A/G magnetic beads for 4 h. The protein amounts were determined using an Enhanced BCA Protein Assay Kit. The normalized protein amounts were subjected to SDS-PAGE and transferred onto polyvinylidene fluoride membranes for Western blotting. The membranes were incubated with specific primary antibodies at 4 °C overnight and then incubated with the appropriate horseradish peroxidase-conjugated secondary antibody for 1 h. Proteins were visualized using Clarity Western ECL Substrate on a ChemiDoc XRSþ system. The expression of proteins was quantified by densitometry using ImageJ software according to three repeated assays and normalized to β-actin levels.

### ChIP assay, real-time PCR

A ChIP assay was performed using SimpleChIP Enzymatic Chromatin IP Kit according to the manufacturer’s instructions. BT-549 cells were cross-linked with 1% formaldehyde and then washed with cold PBS, lysed with the lysis buffer, and then sonicated to produce an average DNA length of 500–1000 bp. Immunoprecipitation was then performed with the indicated antibodies. Purified DNA fragments were analyzed by qPCR using 2×SYBR Green Pro Taq HS Premix on a LightCycler 480 Real-Time system (Roche), and precipitated DNA was calculated as a percentage of input DNA. RNA extraction was performed using TRIzol reagent, and cDNA was prepared using Evo M-MLV RT Master Mix Kit. The primers used for the ChIP assay and qPCR are listed in Supplementary Data 2.

### Enzyme-linked immunosorbent assay for 5-HT and HETEs

MES cells (BT-549 and CAL-120) (10^5^ cells/well) were plated over-night in six-well plates and then treated for 24 h with RSL3, 5-HT or vehicle control for indicated concentration. Five volumes of ice-cold lysis buffer supplemented with protease inhibitor tablets were added to each well. Cell lysates were mechanically dissociated and centrifuged (10,000 × *g* for 15 min at 4 °C), and then diluted 1:1 with calibrator diluent. 5-HT and HETEs levels were then determined by human ELISA kit according to the manufacturer’s instructions. The absorbance was read at 450 nm on a Synergy™ HT Multi-Mode Microplate Reader (Bio-Tek).

### Malondialdehyde assay

A Lipid Peroxidation MDA Assay Kit was employed to assess the level of malondialdehyde (MDA). After homogenization of the collected cells or tissues in 400 µL specialized lysis buffer, the supernatant was collected by sonication and centrifugation. After a 1-hour incubation at 95 °C, a mixture of 600 µL of TBA (thiobarbituric acid) solution and 200 µL of supernatant was applied to determine the absorbance at 532 nm.

### Glutathione peroxidase 4 (GPX4) activity assay

Cells were treated with or without RSL3 and the culture medium and cells were collected for GPX4 activity assays.A Glutathione Peroxidase 4 Activity Assay Kit (Elabscience Biotechnology Co., Ltd.) was used to measure the activity of GPX4 according to the manufacturer’s instructions.

### BODIPY 581/591 C11 assay

C11-BODIPY 581/591 was used to determine the level of lipid ROS (reactive oxygen species). The cells were cultured in confocal dishes, and 1 µM RSL3 was added for 12 h. Fluorescence was detected by confocal microscopy at excitation wavelengths of 565 nm and 488 nm after 1 h of staining with 5 µM BODIPY 581/591 C11 at 37 °C and two washes with PBS. Fluorescence at an emission wavelength of 590 nm represented normal cells, while fluorescence at 510 nm represented oxidized cell membranes.

### Flow cytometry

Tumors were harvested from tumor-bearing mice. Tumor weight was measured before dissociation, and tumors were processed into single-cell suspensions. The antibodies used for flow cytometry are listed in Supplementary Data 2. BD Fix/Permeabilization buffer was used for intracellular staining of IFN-γ and FOXOP3 in CD4+ and CD8+ cells. Data were acquired on a BD Fortessa or a BD FACSCalibur, and analyzed with FlowJo software V10 (Oregon, USA).

### Mitochondrial membrane potential

Cells were treated with RSL3 for 12 h and stained with the JC-1 dye to assess mitochondrial membrane potential. Immunofluorescence was performed to visualize JC-1 fluorescence. The monomeric form of JC-1 exhibits a maximum excitation wavelength of 514 nm and a maximum emission wavelength of 529 nm; whereas the J-aggregates exhibit maximum excitation and emission wavelengths of 585 nm and 590 nm, respectively.

### Transmission electron microscope

Cultured macrophages were fixed in sodium cacodyl atebuffered (0.1 mol/L, pH 7.4) 2.5% glutaraldehyde solution for 2 h, then rinsed (3 × 10 min) in sodium cacodylatebuffered (0.1 mol/L pH 7.4) 7.5% saccharose and postfixed in 1% OsO4 solution for 1 h. After dehydration in an ethanol gradient (70% ethanol for 20 min, 96% ethanol for 20 min, 100% ethanol for 2 × 20 min), samples were embedded in Durcupan ACM and then were stained with uranyl acetate and lead citrate. Sections were examined under an FEI TECNAI spirit (USA) microscope at 100 kV.

### RNA-sequence analysis

RNA was harvested from 1 × 10^6^ cells in triplicate and stored in RNAlater RNA stabilization solution (ThermoFisher Scientific). RNA purification, quantification and qualification, library construction and transcriptome sequencing were performed at Tgene Biotech (Shanghai) Co., Ltd. (Shanghai, China) according to the manufacturer’s instructions. Briefly, RNA was isolated using Trizol reagent. mRNA was purified from total RNA using poly-T oligo-attached magnetic beads. Fragmentation was carried out using divalent cations under elevated temperature in NEBNext. First-strand cDNA was synthesized using a random hexamer primer and M-MuLV Reverse Transcriptase. Second-strand cDNA synthesis was subsequently performed using DNA Polymerase I and RNase H. Remaining overhangs were converted into blunt ends via exonuclease/polymerase activities. After adenylation of the 3’ends of DNA fragments, NEBNext Adaptor with a hairpin loop structure was ligated to prepare for hybridization. In order to select cDNA fragments of preferentially 250~300 bp in length, the library fragments were purified with AMPure XP system (Beckman Coulter, Beverly, USA). Then 3 µL USER Enzyme was used with size-selected, adaptor-ligated cDNA at 37° C for 15 min followed by 5 min at 95 °C before PCR. Then PCR was performed with Phusion High-Fidelity DNA Polymerase, Universal PCR primers and Index (X) Primer. Finally, PCR products were purified (AMPure XP system), and library quality was assessed on the Agilent Bioanalyzer 2100 system. The clustering of the index-coded samples was performed on a cBot Cluster Generation System using TruSeq PE Cluster Kit v3-cBot-HS according to the manufacturer’s instructions. After cluster generation, the library preparations were sequenced on an Illumina Novaseq6000 platform, and 150 bp paired-end reads were generated. After quality control, STAR was used to align clean reads to the reference genome. HTSeq v0.6.0 was used to count the read numbers mapped to each gene. Then the FPKM of each gene was calculated based on the length of the gene and reads count mapped to this gene. We applied the DESeq2 algorithm to filter the differentially expressed genes, after the significant analysis and FDR analysis under the following criteria:log2FC > 1 and *p* value < 0.05.

Differential expression analysis was performed using the DESeq2 R package (v1.39.8) [22], and identified DEGs were sorted by *P*-values. Functional enrichment analysis, including Gene Ontology (GO) biological process (BP) and Kyoto Encyclopedia of Genes and Genomes (KEGG) pathway analyses, was conducted using DAVID with significance set at *p* < 0.05. Additionally, Gene Set Enrichment Analysis (GSEA) was performed to further explore the functional implications of DEGs.

### Metabolites analysis

Targeted cell metabolites analysis was conducted by Tgene Biotech (Shanghai) Co., Ltd. In brief, amino acids were determined using an Ultimate3000 DGLC (ThermoFisher) and an ACCQ-Tag TM ULTRA C18 (100*2.1 mm, 1.8um) liquid chromatography column. Medium and long-chain fatty acids were determined by a 7820A-5977B gas chromatograph-mass spectrometer (Agilent Technologies Inc., CA, USA).

### Cut&Tag analysis

CUT&Tag was performed as previously described [23]. Briefly, 1 × 10^5^ cells were harvested in NE buffer (20 mM HEPES-KOH, pH 7.5, 0.5 mM Spermidine, 10 mM KCl, 0.1% TritonX-100, 10% Glycerol, 1 mM PMSF) and iced for 10 min. ConA beads were pre-washed and resuspended by binding buffer (20 mM HEPES-KOH, pH 7.5, 10 mM KCl, 1 mM CaCl2, 1 mM MnCl2). 10 µL beads were added to each sample and incubated at room temperature for 10 min. The beads were washed with washing buffer (20 mM HEPES-KOH, pH 7.5, 0.5 mM spermidine, 150 mM NaCl, 0.1% BSA) and resuspended in blocking buffer (20 mM HEPES-KOH, pH 7.5, 0.5 mM spermidine, 150 mM NaCl, 0.1% BSA, 2 mM EDTA) at room temperature for 5 min. Primary antibodies (Rabbit monoclonal anti-Histone H3K4me3) were added by 1:100 dilution and incubated at room temperature for 2 h. After being washed with washing buffer, secondary antibodies were added by 1:100 dilution and incubated at room temperature for 30 min. 1.2 µL PA-Tn5 transposomes were added to each sample and incubated at room temperature for 30 min. Beads were resuspended in 30 µL washing buffer with 10 mM MgCl2 and incubated at 37 °C for 1 h. Reactions were stopped by adding 5.5 µL stop buffer (2.25 µL of 0.5 M EDTA, 2.75 µL of 10% SDS and 0.5 µL of 20 mg/ ml Proteinase K) and incubated at 55 °C for 30 min, and then at 70 °C for 20 min to inactivate Proteinase K. 0.9X of VAHTS DNA clean beads were added to each sample to extract the fragmented DNA. DNA was purified using phenol-chloroform-isoamyl alcohol extraction and ethanol precipitation. To amplify libraries, 21 µL DNA was mixed with 2 µL of a universal i5 and a uniquely barcoded i7 primer. A volume of 25 µL NEBNext HiFi 2× PCR Master Mix was added and mixed. The sample was placed in a Thermo cycler with a heated lid using the following cycling conditions: 72 °C for 5 min; 98 °C for 30 s; 14 cycles of 98 °C for 10 s and 63 °C for 30 s; final extension at 72 °C for 1 min and hold at 8 °C. The library fragments were purified with XP beads. The size distribution of libraries was determined by Agilent 4200 TapeStation analysis, and libraries were mixed to achieve equal representation as desired, aiming for a final concentration as recommended by the manufacturer. Sequencing was performed on the Illumina Novaseq 6000 using 150 bp paired-end following the manufacturer’s instructions.

Raw reads of the fastq format were first processed through in-house scripts. All the downstream analyses were based on high-quality clean data. The clean reads were then aligned to reference genome sequences using the BWA program. Using the BAM file generated by uniquely mapped reads as an input file, we employed the MACS2 software for peak calling with a cutoff *q*-value < 0.05. Peaks were annotated using Homer’s annotatePeaks.pl. We counted the annotation results and plotted the distribution results using R. The Homer’s find Motifs Genome.pl tool was used for Motif analysis.

### Dual-luciferase reporter assay

Promoter of MAOA was introduced in to psiCHECKTM-2 reporter (Promega) with endonuclease sites NotI and XhoI, and complementary sequence mutation sites of seed sequence were designed on MAOA to establish MAOA-mutantion (mutantion1, mutantion2, mutantion3). The dual luciferase reporter plasmids MAOA-wt and MAOA-mutantions were co-transfected with QKI or control vector respectively into BT-549 cells. Cells were harvested and lysed 48 h after transfection, and the dual-luciferase reporter assay kit (Beyotime Biotechnology Co., Ltd., Shanghai, China) was used to detected Renilla luciferase assay and firefly luciferase separately. The Renilla luciferase served as internal control. The transfection experiments were performed in triplicate for each plasmid construct.

### Molecular docking

Molecular docking is a commonly used technique to study how small molecules interact with target proteins and evaluate their affinities at particular binding locations. We download molecular structure files of TKIs (Pacritinib, Britinib, Critinib, and Crizotinib) from the PubChem database.The PDB database was used to find and download the molecular structure file of the target protein SLC22A3 (7ZH0). We download molecular structure files for SLC22A3 from the Pubchem database. The downloaded target proteins underwent processing with PyMOL2.3.0 software in order to eliminate water molecules and original ligands. Molecular mechanics optimization of the optimal conformation of all molecules was performed using Chem3D (2020 edition) software, and finally we obtained the optimal conformation with minimal energy.

### SLC22A3 activity inhibition assay

Inhibition of cellular SLC22A3 activity was investigated using the fluorescent SLC22A3 probe DiASP, which is transported into BT-549 and CAL-120 cells in a saturable manner (Km = 24.8 µM) as the previous study showed [24, 25]. Briefly, cells were incubated with 10 µM DiASP for 5 min at 37 °C, in the absence or presence of the reference SLC22A3 inhibitor corticosterone or of TKIs, in the transport assay medium previously described [26]. After washing with phosphate-buffered saline (PBS), intracellular accumulation of the dye was determined by spectrofluorimetry, using a SpectraMax Gemini SX spectrofluorometer (Molecular Devices); excitation and emission wavelengths were 485 nm and 607 nm, respectively. Data were finally normalized to total protein content, determined by the Bradford’s method [27]. They were expressed as fluorescence arbitrary unit (FAU)/mg protein or as percentages of OCT3 activity or of OCT3 activity inhibition according to the Eqs. (1) or (2), respectively:

1 $$\% {\rm{SLC}}22{\rm{A}}3\,{\rm{activity}}=\frac{\left(\left[{{\rm{DiASP}}}_{{\rm{TKI}}}\right]-\left[{{\rm{DiASP}}}_{{\rm{corticosterone}}}\right]\right)\times 100}{\left(\left[{{\rm{DiASP}}}_{{\rm{Control}}}\right]-\left[{{\rm{DiASP}}}_{{\rm{corticosterone}}}\right]\right)}$$

2 $$\% {\rm{SLC}}22{\rm{A}}3\,{\rm{activity}}\,{\rm{inhibition}}=100 \% - \% {\rm{SLC}}22{\rm{A}}3\,{\rm{activity}}$$

with [DiASPTKI] = DiASP concentration in the presence of a defined concentration of TKI, [DiASP_Corticosterone_] =DiASP concentration in the presence of 100 µM corticosterone and [DiASP_Control_] = DiASP concentration in control cells not exposed to TKI or corticosterone. Half maximal inhibitory concentration (IC50) for TKIs toward SLC22A3 activity was calculated using Prism 8.4.2 software (GraphPad Software).

### Tissue dissociation and organoid culture

We collected TNBC specimens and classified them, screening for MES-positive tumors based on the immunohistochemical (IHC) signature: SLC22A3⁺/CD8⁻/FOXC⁻/AR⁻/DCLK1⁺.To digest tissue fragments into cell cluster, tissue digest solution was prepared using 10 mL of DMEM/F12 medium supplemented with 0.1 mg/mL of DNase, 0.018 g of collagenase, and 10 mM of Y27632. Fresh tissue was cut into 1–3 mm^3^ pieces and digested on a shaker at 37 °C 140 rpm for 0.5–1 h. After digestion, the samples were filtrated through a 70 μm filter. The isolated cell mass suspension was centrifuged at 400 × *g* for 4 min, washed once with DMEM/F12 medium, and centrifuged again at 400 × *g* for 4 min. If there were too many red blood cells in the pellet, the cell lysates would be incubated with Red Blood Cell Lysis Buffer (Servicebio; Wuhan, China) for 5 min, and the supernatant was removed by centrifugation at 1000 rpm. The cell clusters were resuspended in 4 °C Matrigel and seeded at 30 µL/well in prewarmed 24-well plates. The suspension was incubated for 30 min in an incubator at 37 °C with 5% CO_2_. Next, 500 μL BC organoid medium was added to each well. The organoids were passaged every 1–3 weeks following a previously reported protocol. Briefly, the medium was discarded, and 500 μL of cell recovery solution was added to each well. The plates were incubated in a precooled culture shaker at 4 °C for 30 min to completely dissolve the matrix gel. Then, the organoid suspension was centrifuged at 4 °C at 400 × *g*/min for 5 min. Homogenize the organoids 20 times with pipette tips; TrypLE is used to digest large organoids. The supernatant was discarded, and the organoids were resuspended in a matrix gel precooled at 4 °C and transferred to a new 24-well plate with 2000–5000 cells per well.

### Synergistic effect analysis

BT-549 cells were treated with a series of concentrations (0, 5, 10, 20, and 40 μM) of each TKI alone, cisplatin or paclitaxel alone, or in combination. After 12 h of incubation, 10 μL of CCK-8 solution was added to each well, and the plates were incubated for an additional 2 h at 37 °C. The absorbance was measured at a wavelength of 450 nm using a microplate reader. The drug-drug interaction (synergism, additivity, or antagonism) was quantitatively determined by calculating the Combination Index (CI) using the Chou-Talalay method. According to the standard interpretation, a CI < 1 indicates synergism, CI = 1 indicates additivity, and CI > 1 indicates antagonism.

### In vivo tumorigenesis assay

To explore the function of SLC22A3 on the immune resistance of tumors in vivo, a 4T1 cell-bearing murine model and xenograft tumor studies were employed. In 4T1 cell-bearing murine model, 1 × 10^5^ 4T1 cells in 100 µL PBS and Matrigel (47743-720, Corning) mixture (1:1) were injected into the mammary fat pads. Then 7 days later, five intraperitoneal injections with 10 mg/kg RSL3 (every other day) were performed. Vehicle control was also injected as a negative control. Meanwhile, mice were also treated with ceritinib or paritinib (10 mg/kg, oral gavage, every other day) or administered the vehicle control.

Xenograft tumor studies were conducted as previously described [28, 29]. In short, CB-17/SCID female mice were allowed a period of adaptation in a sterile and pathogen-free environment with food and water. BT-549-tRFP cells were harvested in the exponential growth phase using a PBS/EDTA solution and washed. Viable cells (5 × 10^6^) in 50 µL of sterile PBS suspension were mixed with 100 µL reduced growth factor Matrigel (BD Biosciences) and injected bilaterally into the inguinal mammary fat pad. On day three post cell injection, mice were randomized into treatment groups of five mice each: (vehicle control or 10 mg/kg panobinostat). Beginning on day 14 post cell injection, animals received intraperitoneal (i.p.) injections of the corresponding drug treatment on a five-day and two-day off schedule for 28 days [30]. Tumor size was measured with a digital caliper and calculated using the formula 4/3πLS^2^ (L = larger radius, S = smaller radius). At necropsy, animals were euthanized. Tumors were removed and snap frozen or fixed in 10% formalin for future analysis. The study was approved by the Air Force Medical University Experimental Animal Ethics Committee (no 250106-8).

All procedures involving animals were conducted in compliance with guidelines established by the Forth Military Medical University Committee. The facilities and laboratory animal programs of The Forth Military Medical University are accredited by the Association for the Assessment and accreditation of laboratory animal care.

### Statistical analyses

Significance of variations was assessed using independent *t*-tests or one-way ANOVA with Tukey’s post-test. A *p*-value below 0.05 (two-tailed) was deemed to be statistically significant, suggesting the existence of significant results. We analyzed the categorical data statistically using the Fisher's exact probability method. SPSS was used for statistical analysis and *p* < 0.05 was considered statistically significant. All statistical analyses were performed with R software (http://www.R-project.org, version 3.5.2) or GraphPad Prism software (version 9.0).

## Results

### Identification of serotonin transporter SLC22A3 as a maker of mesenchymal subtype TNBC

**Fig. 1:**
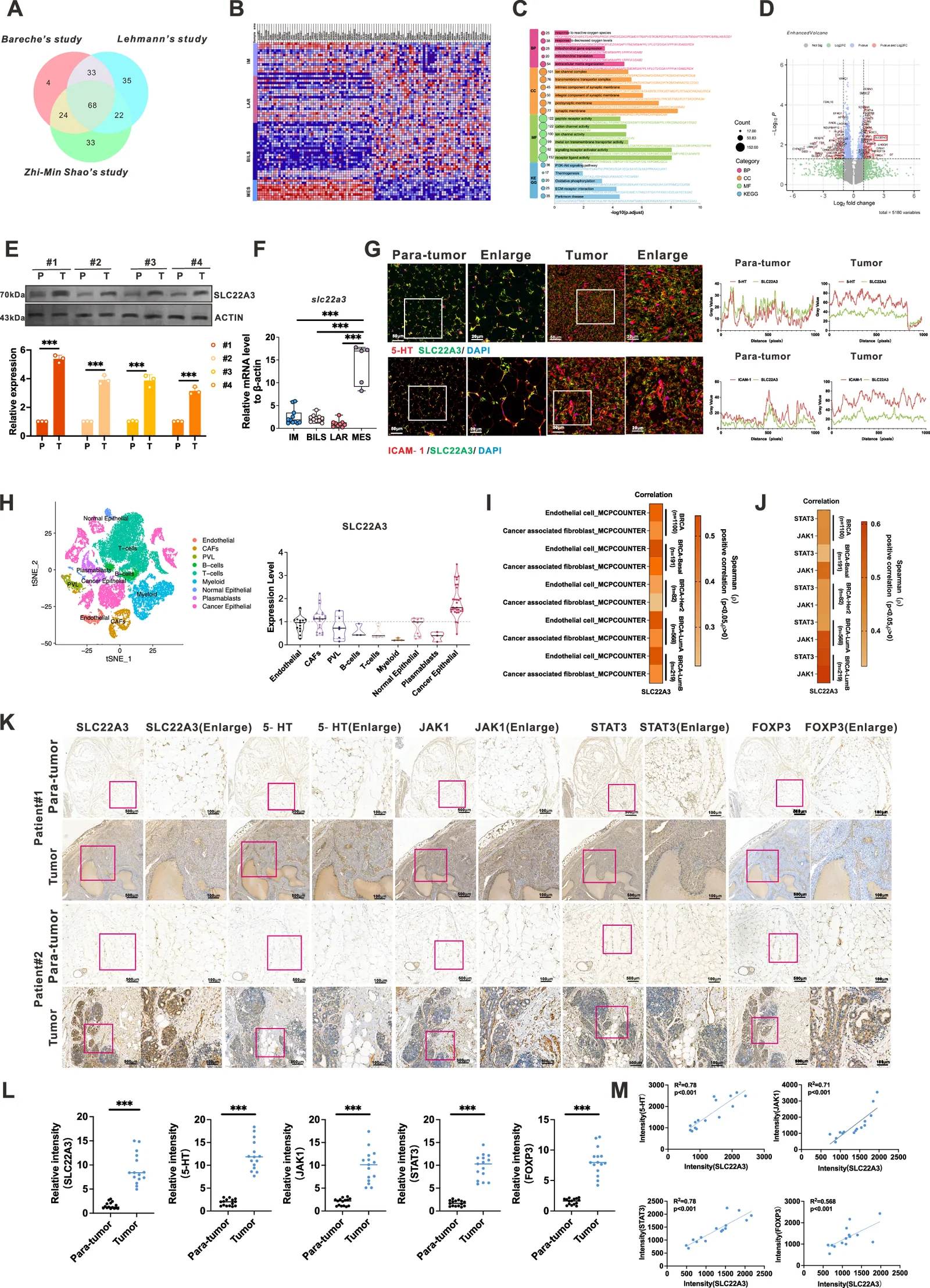
SLC22A3 is a maker of mesenchymal subtype TNBC. **A** Venn diagram analysis the common subtype samples extracted from different studies. **B** Heatmap analyzed the different genes between distinct four groups. **C** The up-regulation genes were analyzed by Biological process (BP), Cellular component (CC) and Molecular function (MF). **D** Volcano analysis of the different genes (MES versus the other subtypes). **E** The expression of SLC22A3 was analyzed in tumor tissue samples from MES TNBC patients and the corresponding adjacent non-cancerous (para-tumor) tissues (*n* = 4). **F** The mRNA expression of SLC22A3 was analyzed in different subtypes tumor tissue samples. **G** The co-localization and expression of SLC22A3, ICAM-1 and 5-HT in MES tumor tissue and the corresponding adjacent non-cancerous (para-tumor) tissues were analyzed by immunofluorescence. **H** Single cell sample (GSE176078) analyzed the expression of SLC22A3. TIMER2.0 platform analyzed the correlation of SLC22A3 with subtypes (Basal, Her2, LumA and LumB) (**I**), and JAK1, STAT3 expression (**J**), positive correlation: *p* < 0.05, ρ > 0. **K**–**M** Immunohistochemical analysis of the expression levels of SLC22A3, JAK1, STAT3, 5-HT and FOXP3 in MES subtype tumor tissues and adjacent non-cancerous (para-tumor) tissues. Quantification of intensity of protein (SLC22A3, JAK1, STAT3,5-HT and FOXP3) expression in IHC (**L**), and relationship analysis between SLC22A3 and JAK1, STAT3,5-HT, FOXP3 respectively (**M**), Data are presented as mean ± SEM. ***P < 0.001 by independent sample *t* test (**E**, **F**, **L**).

To systematically identified the characteristic of mesenchymal subtype TNBC, we firstly screened the common subtype samples from TGCA, which used in the previous studies [3, 5, 6]. By using the intersection method, we selected TNBC subtype samples with consistent classifications from three laboratories (Bareche, Shao Zhimin, and Lehmann) for subsequent analysis (totaling 68 cases). The common samples include 10 cases of MES subtype, 24 cases of BILS subtype, 12 cases of LAR subtype, and 22 cases of IM subtype (Fig. 1A and Supplementary Data 1). Further gene expression analysis revealed that MES subtype showed distinguished gene pattern, compared with other subtypes (Fig. 1B). By using Cellular Component (CC) analysis, we found an upregulation of gene expression related to the synaptic membrane and postsynaptic membrane (Fig. 1C). Cell Component (CC) analysis revealed that upregulated genes were predominantly enriched in neural pathways (intrinsic component of synaptic membrane, integral component of synaptic membrane, postsynaptic membrane and synaptic membrane). Among these, solute carrier (SLC) family genes, which are known to play crucial roles in neurotransmitter transport and neuronal homeostasis, showed significant enrichment. SLC22A3 (Organic Cation Transporter 3, OCT3) is a well - characterized member of this family with established functions in the central nervous system. As the volcano results showed, SLC22A3 expression was increased dramatically in MES samples.The expression of DCLK1 was also detected in MES subtype, consistence with the previous studies [7] (Fig. 1D).

We further investigated the SLC22A3 expression in TNBC samples. The expression of SLC22A3 was increased in tumor tissue samples from MES TNBC patients, compared with the corresponding adjacent non-cancerous (para-tumor) tissues (Fig. 1E). Moreover, after classified into subtypes according to the previously reported IHC classification method [7], we found that the expression of *slc22a3* mRNA expression in MES subtype was higher than other subtypes in tumor tissues (Fig. 1F).

Considering the role of SLC22A3 in 5-HT transporting, we found the co-localization and increased expression of SLC22A3 and 5-HT in MES sample by immunofluorescence. Moreover, SLC22A3 was co-localized with ICAM-1, an important maker of endothelial cell. Then we confirmed that SLC22A3 expression by single cell sample from TNBC patient (GSE176078). The results showed that the SLC22A3 mainly expressed in cancer epithelial cells (Fig. 1H). TIMER2.0 platform analysis provided more robust evidence that the expression of SLC22A3 was positively correlated with endothelial cells and cancer-associated fibroblast in breast cancer in subtypes (Basal, Her2, LumA and LumB) (Fig. 1I).

MES subtype exhibited with distinct characteristics of tumor stem cells and immune desert-like phenotype, with high expression of JAK1-STAT3 pathway, and less immune cells infiltration, as reported in previous study [6]. Both TIMER2.0 platform analysis of breast cancer subtypes (Basal, Her2, LumA and LumB) and IHC of MES samples confirmed that SLC22A3 expression was positively correlation with JAK1, STAT3 and FOXP3 expression (Fig. 1J–M). These data collectively confirmed the higher expression of SLC22A3 in MES subtype and being mainly expressed in cancer epithelial cells.

### SLC22A3 silencing inhibited 5-HT mediated anti-ferroptosis

**Fig. 2:**
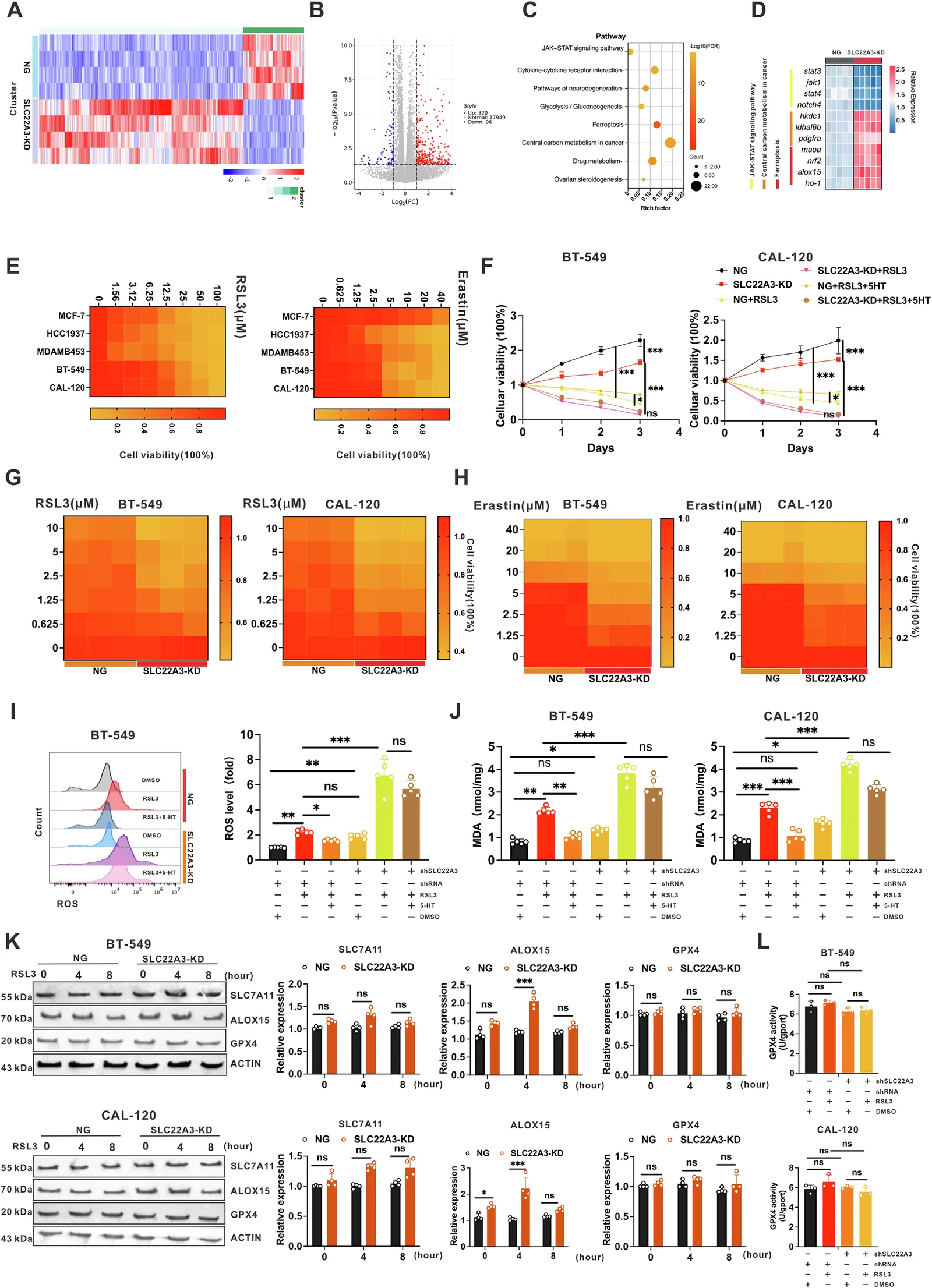
SLC22A3 deficiency increased the sensitivity to RSL3 and Erasin in MES subtype cells. **A** RNA-sequencing analyzed the difference genes between the negative control (NG) and SLC22A3 deficiency(SLC22A3-KD) BT-549 cells. **B** Volcano plot illustrating the gene expression profiles (SLC22A3-KD versus NG group). **C** KEGG analyzed the different genes from RNA-sequencing enriched pathway. **D** The expression of genes identified in the KEGG enrichment analysis was validated by quantitative real-time PCR (qRT-PCR) (*n* = 5). **E** The sensitivity of different cell subtypes (non-TNBC: MCF-7, Basal:HCC1937 LAR:MDAMB453, MES: BT-549 and CAL-120) to inducer of ferroptosis (RSL3 and Erasin). **F** CCK8 analyzed cell viability of BT-549 and CAL-120 under RSL3 (10 μM) or 5-HT (5 μM) treatment for 8 h, *n* = 3. **G**, **H** The sensitivity of negative control(NG) and SLC22A3 knockdown (SLC22A3-KD) MES cells (BT-549 and CAL-120) to RSL3 (**e**) (*n* = 4) and Erasin (**f**). **I** The level of ROS in NG and SLC22A3-KD BT-549 cells after under RSL3(10 μM) or 5-HT (5 μM) treatment for 8 h were analyzed by Flow Cytometry, *n* = 5. **J** The MDA level of in NG and SLC22A3-KD MES cells (BT-549 and CAL-120) after under RSL3(10 μM) or 5-HT (5 μM) treatment for 12 h were analyzed by ELSIA, *n* = 5. **K** The expression of ferroptosis-related pathways (SLC7A11, ALOX15, GPX4) in NG and SLC22A3-KD MES cells (BT-549 and CAL-120) was analyzed using western blot after treatment with RSL3 (10 μM) for 8 h, *n* = 4. **L** The GPX4 activity was measured by ELISA after RSL3(10 μM) treatment in NG and SLC22A3-KD MES cells (BT-549 and CAL-120). Data are presented as mean ± SEM. *P < 0.05, **P < 0.01, ***P < 0.001 by independent sample *t* test (**C**, **E**, **F**, **I**), 1-way ANOVA with Tukey’s posthoc test (**G**, **H**) or 2-way ANOVA (**D**).

To further investigate the role of SLC22A3 in mesenchymal (MES) breast cancer cells, we found that the mRNA and protein level of SLC22A3 in MES breast cancer cells were higher than non-MES cells (Supplementary Fig. S1A, B). Among of MES cells, the BT-549 and CAL-120 cell lines were used as representative models. SLC22A3 was knocked-down in both BT-549 and CAL-120, which was not influence the expression of SLC22A1 and SLC22A2 (SLC22A3-KD, Supplementary Fig. S1C–E). We found that SLC22A3 knockdown significantly decreased the 5-HT level in MES cells, even when 5-HT was supplemented (Supplementary Fig. S1F). RNA-sequencing results of BT-549 cells revealed significant changes in gene expression following SLC22A3 knockdown (Fig. 2A). Volcano analysis reveled 320 genes were up-regulated and 96 genes down-regulated (Fig. 2B), and Deferentially expressed genes following SLC22A3 depletion were significantly enriched in ferroptosis, central carbon metabolism, cancer and drug metabolism pathway (Fig. 2C). Specifically, genes associated with the JAK-STAT signaling pathway (*stat3, jak1,stat4*) were downregulated, conversely, those involved in ferroptosis (*maoa, nrf2, alox15, ho-1*) and central carbon metabolism in cancer (*hkdc, pdgfra*) were upregulated (Fig. 2D). MES cells resisted to ferroptosis with lower level of peroxidation production in previous study [15]. We found that MES cell lines (BT-549 and CAL-120) were resistant to inducers of ferroptosis (RSL3 and Erasin), when compared with other subtypes (non-TNBC, BLIS and LAR) (Fig. 2E). We speculated that 5-HT might mediated the anti-ferroptosis effect in MES cell lines. With 5-HT treatment at difference doses, both BT-549 and CAL-120 cells were more resistant to ferroptosis induced by RSL3 (Supplementary Fig. S1G). However, 5-HT treatment promoted the growth and inhibited the RSL3 sensitivity of MES cell lines, but this effect was absent in the SLC22A3-KD cells (Fig. 2F). SLC22A3 deficiency in MES cells significantly enhanced the sensitivity to ferroptosis inducers (RSL3 and Erasin) (Fig. 2G, H), and overexpression of SLC22A3 in non - MES cell lines (HCC1937, MDMB453) augmented the resistance to ferroptosis (Supplementary Fig. S1H–J). Meanwhile, the levels of ROS (Reactive Oxygen Species) and MDA (Malondialdehyde) were elevated following RSL3 treatment and were further increased in the SLC22A3-KD cells. While 5HT was able to reduce RSL3-induced ROS and MDA levels, this effect was not fully observed in the SLC22A3-KD cells (Fig. 2I, J). Under the stimulation of RSL3, the expression of ferroptosis-related proteins (SLC7A11 and GPX4) and GPX4 activity remained unchanged in MES cells, as well as in the SLC22A3-KD cells. However, the expression of ALOX15 was higher in SLC22A3-KD cells compared with the negative controls (Fig. 2K). At the same time, No significant alteration in GPX4 enzymatic activity was observed upon SLC22 ablation (Fig. 2L). The results indicated that SLC22A3’s role in ferroptosis is partly dependent on the transport of serotonin (5-HT), which acts as an antioxidant agent against oxidative stress [31, 32].

**Fig. 3:**
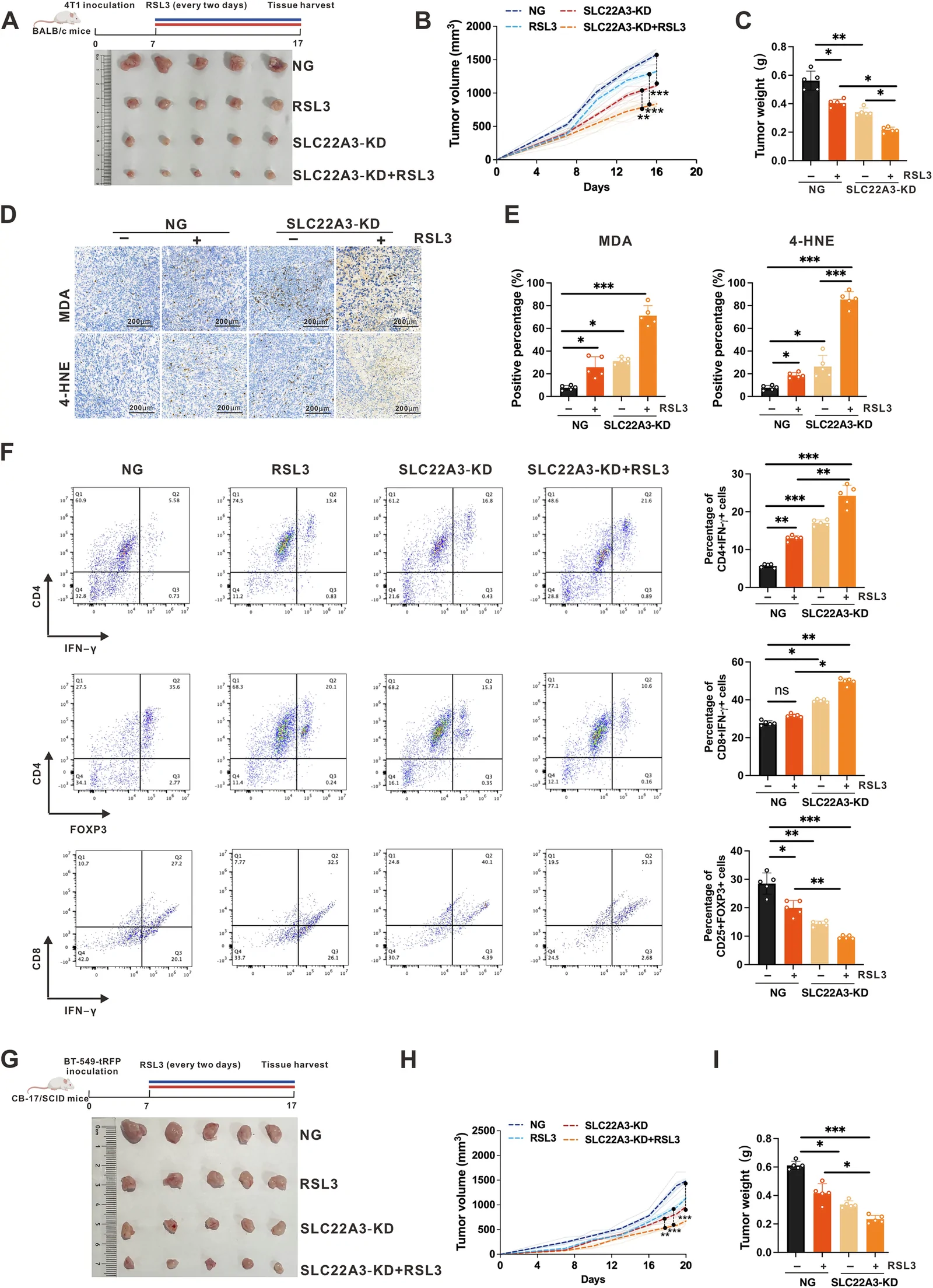
knockdown of SLC22A3 increased the susceptibility to RSL3 in vivo. **A**–**C** NG cells and SLC22A3-KD 4T1 cells were engrafted into mammary fat pads of BALB/c mice (*n* = 5/group), followed with RSL3 treatment (40 mg/kg) intraperitoneally (i.p.) or Negative control (NG) (1:20 DMSO in normal saline) every two days from day 7. The Tumor size (**A**, **B**) and tumor volume (**C**) in each group were measured. The expression of MDA and 4NHE were measured in tumor tissues from different groups (**D**), *n* = 5. The quantification was analyzed from five views (**E**). **F** The population of CD4 + IFN-γ+, CD8 + IFN-γ+ and CD4 + FOXP3+ cells in tumor tissues from different groups were analyzed by Flow Cytometry, *n* = 5. **G**–**I** Female, CB-17/SCID mice (*n* = 5/group) were injected with BT-549-tRFP cells into the inguinal mammary fat pad. On day 7, mice were treated intraperitoneally (i.p.) with RSL3 (40 mg/kg) or Negative control (NG) (1:20 DMSO in normal saline) every two days (**G**). The tumor volume (**H**) and weight were detected (**I**). Data are presented as mean ± SEM. *P < 0.05, **P < 0.01, ***P < 0.001 by 1-way ANOVA with Tukey’s posthoc test (**B**–**I**).

Additionally, we assessed the therapeutic efficacy of inhibiting SLC22A3 and combination with RSL3 treatment. SLC22A3-KD 4T1 cells were engrafted into mammary fat pads of BALB/c mice, which were subsequently treated with RSL3. The results indicated that the knockdown of SLC22A3 inhibited tumor growth in vivo (Fig. 3A, B), and reduced tumor weight (Fig. 3C). Meanwhile, compared with the negative controls (NG), the expression of MDA and 4-HNE were increased in SLC22A3 knockdown tumor tissues and were further elevated following RSL3 treatment (Fig. 3D, E). Considering the damage-associated molecular patterns (DAMPs) released by ferroptotic tumor cells, thereby activating adaptive immunity and enhancing anti-tumor immunity [22]. We found the population of CD4^+^IFN-γ^+^ and CD8 ^+^IFN-γ^+^ cells were increased in SLC22A3-KD group and further elevated with RSL3 treatment, compared with the NG group. Conversely, the population of Treg cells (CD4^+^FOXP3^+^) in SLC22A3-KD group were decreased (Fig. 3F). Moreover, immunocompromised female mice were orthotopically inoculated with NG or SLC22A3-KD BT-549 cells and treated with RSL-3 or vehicle control (Fig. 3G). Similarly, knockdown of SLC22A3 significantly resulted in decreases in tumor volume (Fig. 3H) and tumor weight (Fig. 3I), and promoted RSL3 treatment efficiency in vivo.

### SLC22A3 knockdown blockaded the accumulation of peroxidized phospholipids

**Fig. 4:**
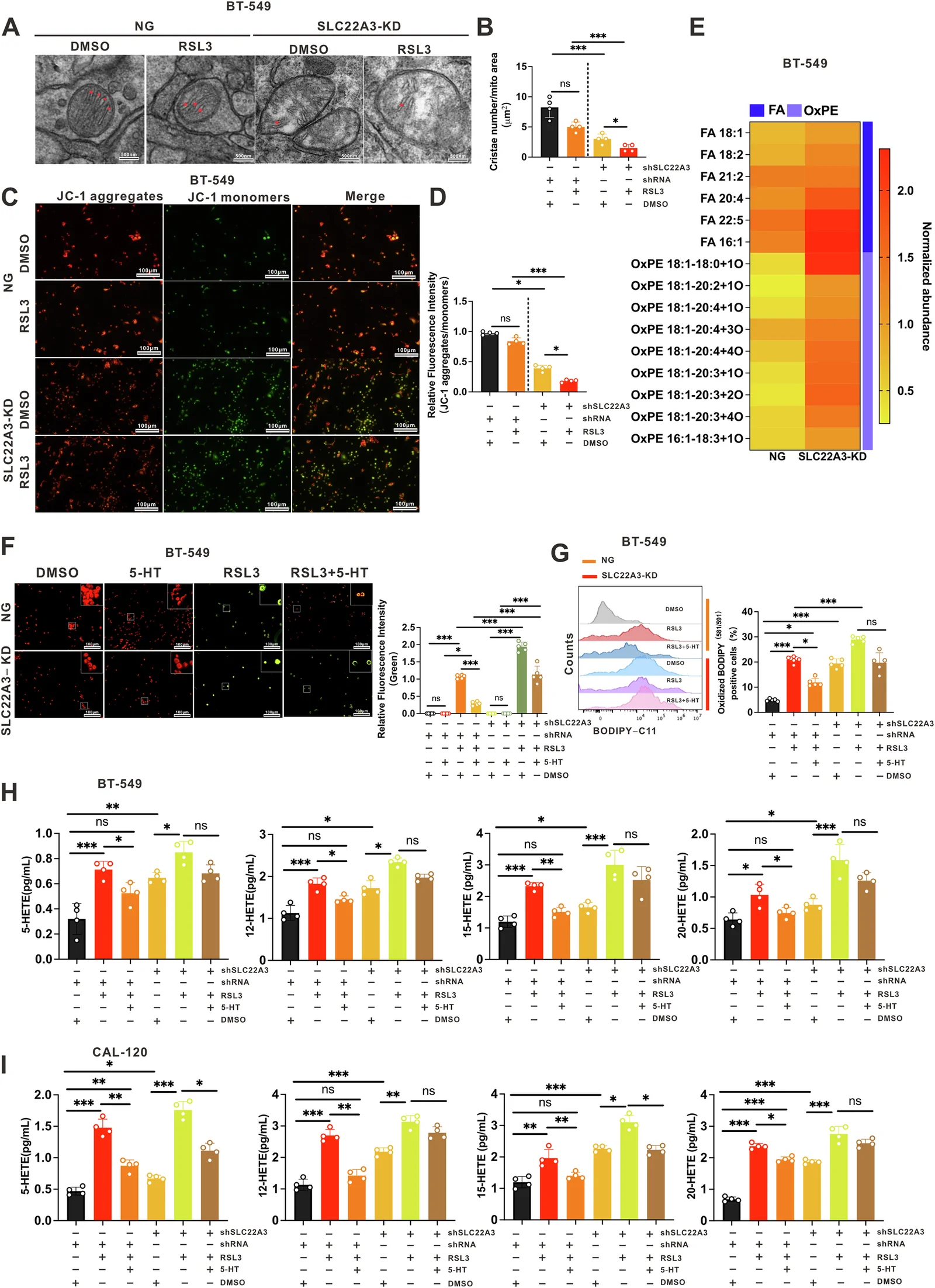
Accumulation of peroxidized phospholipids was observed in SLC22A3 deficiency cells. **A**, **B** Electron microscopy images showing mitochondrial morphology. The mitochondrial cristae are indicated by red arrowheads (**A**). Quantification of crista number per unit mitochondrial area (**B**). **B**–**D** Mitochondrial membrane potential was assessed using JC-1 staining (**C**). The membrane potential was quantified as the ratio of aggregates to monomers (**D**). **E** The metabolites analysis of FA and OxPE in NG and SLC22A3-KD BT-549 cells. **F**, **G** The lipid ROS accumulation in BT-549 cells after RSL3(10 μM) and 5-HT (5 μM) treatment for 8 h and stained with BODIPY-581/591, followed with detection by immunofluorescence (**F**), and flow cytometry (**G**), *n* = 5, respectively. The quantification of relative fluorescence intensity of cells in five fields was analyzed (**F**, right). The Oxidized BODIPY positive cells were analyzed (**G**, right). **H**, **I** The level of HETEs (5-HETE, 11-HETE, 12-HETE, 15-HETE) in BT-549 (**D**) and CAL-120 (**E**) after RSL3(10 μM) and 5-HT (5 μM) treatment for 8 h were analyzed by ELISA, *n* = 4. Data are presented as mean ± SEM. *P < 0.05, **P < 0.01, ***P < 0.001 by independent sample *t* test (**B**, **D**, **E**), 1-way ANOVA with Tukey’s posthoc test (**G**, **H**).

Next, we investigated the impact of SLC22A3 on mitochondria and metabolism in MES cells following RSL3 treatment. A decrease in mitochondrial cristae number in BT-549 cells was detected after RSL3 treatment, albeit without statistical significance. However, SLC22A3 deficiency promoted mitochondrial swelling, which was further exacerbated upon RSL3 treatment, accompanied with mitochondrial cristae reduction (Fig. 4A, B). Similarly, compared with the NG group, SLC22A3 deficiency decreased mitochondria membrane potential, and this reduction was exacerbated by RSL3 treatment (Fig. 4C, D).

Oxidative reactions produce oxidized phospholipids (Oxidized Phosphatidylethanolamine, OxPE), which are less stable and prone to forming lipid peroxides, accelerating ferroptosis. Therefore, we further investigated the level of metabolites (fatty acids and phospholipid peroxidation products) after SLC22A3 knockdown. The results showed that after SLC22A3 deletion, the expression levels of polyunsaturated fatty acids (FA 18:2, FA 21:2, FA 20:4, FA 22:5) and phospholipid peroxides (OxPE 18:1−18:0 + 1O18:1 − 20:2 + 1O, 18:1 − 20:4 + 1O, 18:1 − 20:3 + 2O) were significantly increased, compared with NG cells (Fig. 4E).

We detected the effect of SLC22A3 on lipid peroxidation in the presence of RSL3 or 5-HT. BODIPY-581/591 staining indicated that lipid ROS accumulation was markedly increased in the SLC22A3-KD cells after RLS3 treatment. Although 5-HT supplement inhibited the lipid ROS level, this effect was abolished in SLC22A3-KD cells (Fig. 4F, G). Phospholipids are the main components of cell membranes, containing phosphate groups and fatty acids.

Arachidonic acid (AA) is a prevalent polyunsaturated fatty acid (PUFA) that undergoes oxidative metabolism by enzymes such as lipoxygenases (LOX), cyclooxygenases, and cytochrome P450, resulting in a complex mixture of metabolites from lipid peroxidation. The primary products initially formed in these reactions are hydroperoxyeicosatetraenoic acid (HDETE) and hydroxy-eicosatetraenoic acid (HETE). Members of the LOX family, including 5-LOX, 8-LOX, 12-LOX, and 15-LOX, generate metabolites such as 5-HETE, 11-HETE, and 15-HETE. We found that SLC22A3 deficiency in MES cells increased the levels of HETEs, including 5-HETE, 11-HETE, 12-HETE and 15-HETE. Consistence with the previous data, 5-HT could not inhibit the HETEs production in SLC22A3 deficiency cells (Fig. 4H, I).

### MAOA upregulation abrogated the protective effect of 5-HT

**Fig. 5:**
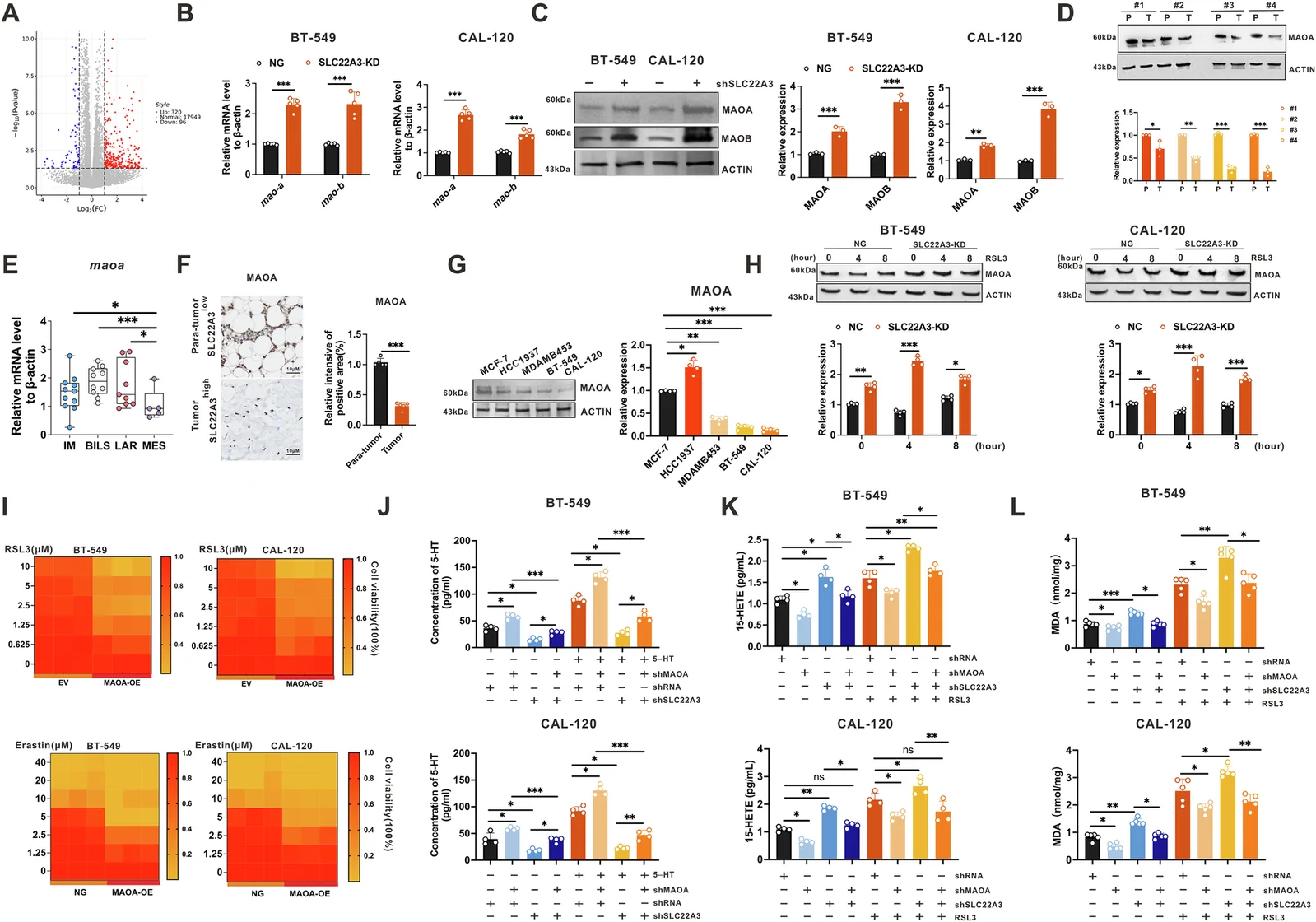
MAOA upregulation abrogated the protective effect of 5-HT. **A** The volcano revealed that different genes between NG and SLC22A3-KD cells in RNA-sequencing analysis, *p* < 0.05 and fold change (FC) > 2. **B**, **C** The mRNA and protein expression of MAOA and MAOB were analyzed by RT-PCR (*n* = 5) and western bolt (*n* = 3). **D** The MAOA expression were analyzed by western blot in TNBC patient tissues and corresponding adjacent non-cancerous (para-tumor) tissues (*n* = 3). **E** The mRNA expression of maoa in different subtypes was analyzed by RT-PCR, **F** The expression of MAOA was analyzed by immunohistochemistry in TNBC tumor tissues (patient SLC22A3^high)^ and corresponding adjacent non-cancerous (para-tumor) tissues (normal, SLC22A3^low^), *n* = 5. **G** The MAOA and MAOB expression in different subtypes cell line were analyzed by western bolt, *n* = 4. **H** The MAOA expression in BT-549 and CAL-120 cells were analyzed by western bolt after RSL3(10 μM) treatment for indicated time, *n* = 4. **I** The sensitivity of Negative controls (NG) and MAOA overexpression BT-549 and CAL-120 cells to inducer of ferroptosis (RSL3 and Erasin), *n* = 4. **J** The NG and SLC22A3-KD cells were knockdown with MAOA,followed with 5-HT (5 μM) treatment. The concentration of 5-HT was analyzed by ELISA, *n* = 4. **K**, **L** The NG and SLC22A3-KD cells were knockdown with MAOA, followed with RSL-3 (10 μM) treatment. The concentration of 15-HETE (*n* = 4) and MDA (*n* = 5) were analyzed by ELISA. Data are presented as mean ± SEM. *P < 0.05, **P < 0.01, ***P < 0.001 by independent sample *t* test (**B**–**D**, **F**–**I**), 1-way ANOVA with Tukey’s posthoc test (**G** and **H**) or 2-way ANOVA (**E**, **J**–**L**).

To discover the potential mechanism of SLC22A3-regulated in ferroptosis, RNA-sequencing revealed that 320 genes were upregulated and 96 genes were downregulated after SLC22A3 silenced (Fig. 5A). We noticed that the expression of MAOA and MAOB were significantly increased after SLC22A3 knockdown. Monoamine oxidase family, including maoa and maob, was involved in the degradation of monoamine neurotransmitters, such as dopamine, serotonin and norepinephrine. We verified that the MAOA and MAOB expression were increased in mRNA and protein level (Fig. 5B, C). While MAOA and MAOB share similar functions, they differ in their substrates. MAOA primarily metabolizes substances like serotonin, norepinephrine (NE), dopamine (DA), and phenylethylamine. On the other hand, MAOB primarily metabolizes dopamine (DA), phenylethylamine, and phenylalanine. The MAOA expression was lower in TNBC tumor tissue compared to the corresponding adjacent non-cancerous (para-tumor) tissues (Fig. 5D). And the expression of *maoa* mRNA was lower in MES samples, compared with other subtypes (Fig. 5E). Similarly, we found that lower expression of MAOA was observed in SLC22A3^high^ TNBC samples, compared with the adjacent non-cancerous (Normal SLC22A3^low^) samples (Fig. 5F). The MES cells (BT-549 and CAL120) exhibited lower expression of MAOA than other subtypes (Fig. 5G). Upon stimulation with RSL3, the expression of MAOA in MES cells was increased in SLC22A3-KD cells compared to the negative controls (Fig. 5H).

To investigate the effect of MAOA-mediated 5-HT elimination in ferroptosis, we verified the sensitivity to RSL3 in MAOA-overexpressed cells (Supplementary Fig. S2A, B). The results indicated that sensitivity to RSL3 was enhanced in both MAOA-overexpressed BT-549 and CAL-120 cells (Fig. 5I). Accordingly, MAOA-silenced cells greatly increased the level of 5-HT and decreased the 15-HETE and MDA in MES cells, as well as restored the resistance to RSL3 (Fig. 5J–L).

### Suppression of histone serotonylation inhibited sirt1 expression in SLC22A3 knockdown cells

**Fig. 6:**
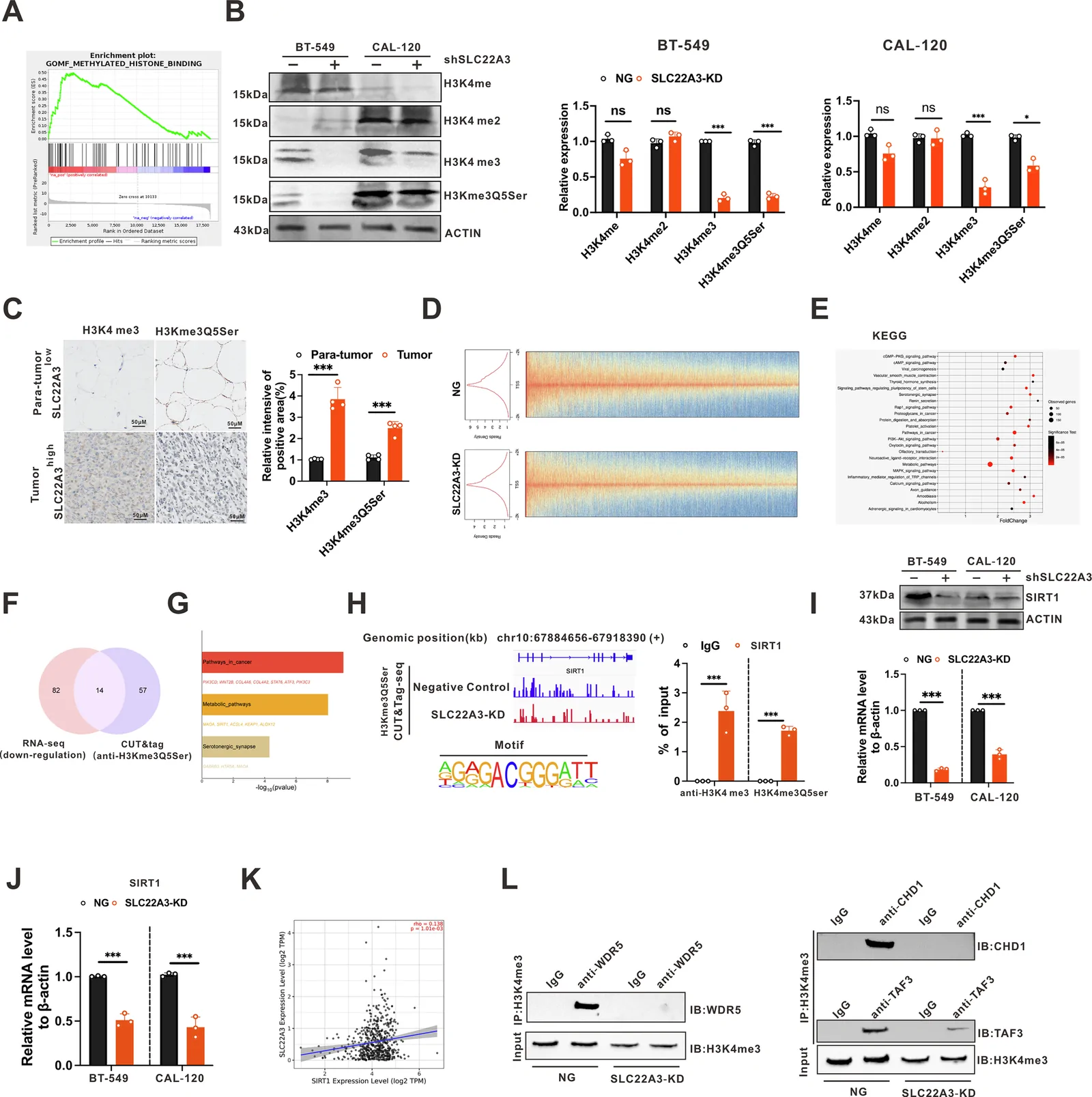
SLC22A3 silencing suppressed histone serotonylation mediated SIRT1 expression. **A** The GESA analysis revealed the different genes in RNA-sequencing enriched in methylated histone-binding function. **B** The expression of H3K4me, H3K4me2, H3K4me3 and H3K4me3Q5Ser in NG and SLC22A3-KD BT-549 cells, *n* = 3. **C** IHC analysis of H3K4me3 and H3K4me3Q5Ser in MES subtype tumor tissues, compared with adjacent non-cancerous (para-tumor) tissues, *n* = 5. **D** CUT&Tag of H3K4me3 within a 3 kb region upstream and downstream of the Transcription Start Site (TSS) in NG and SLC22A3-KD BT-549 cells. **E** CUT-Tag analyzed the different H3K4me3Q5Ser antibody binding DNAs between NG and SLC22A3-KD BT-549 cells, which was enriched in KEGG pathway. **F** The intersection of decreased mRNA and H3K4me3Q5Ser binding DNA in NG versus SLC22A3-KD BT-549 cells. **G** KEGG analysis revealed that the overlapping genes. **H** CHIP-PCR analyzed the H3K4me3 and H3K4me3Q5Ser binding with SIRT1 promoter, *n* = 3. **I**, **J** After SLC22A3 was knockdown, the protein level (**F**) and mRNA level (**G**) were analyzed by western blot and RT-PCR, respectively, *n* = 3. **K** TIMER 2.0 platform analyzed the correlation of SIRT1 and SLC22A3 in tissues from TNBC. **L** Co-inmunoprecipitation analyzed the interaction of H3K4me3 with WDR5, CHD1 and TAF3, respectively.Data are presented as mean ± SEM. *P < 0.05, **P < 0.01, ***P < 0.001 by independent sample *t* test (**B**, **C**, **G**–**I**).

Serotonylation of glutamine is a type of histone post-translational modification, which occurs at position 5 (Q5ser) on histone H3 in organisms that produce serotonin. Previous research has shown that TGM2 (transglutaminase 2) serotonylates histone H3 tri-methylated lysine 4 (H3K4me3)-marked nucleosomes, leading to the presence of combinatorial H3K4me3Q5ser [33]. Interestingly, loss of astrocytic SLC22A3 reduced serotonin levels in astrocytes, resulting in decreasing of histone serotonylation [17]. However, the effect of SLC22A3 mediated serotonylation in transcriptional and epigenomic responses was unclear. Compared with control cells, GSEA(Gene Set Enrichment Analysis) analysis revealed that the expression of methylated histone-binding-related genes were changed in SLC22A3 knockdown cells (Fig. 6A). The results showed that the expression of H3K4me and H3K4me2 were not changed, but the expression of H3K4me3 and H3K4me3Q5Ser were decreased after SLC22A3 knockdown (Fig. 6B). IHC analysis of clinical samples from TNBC (Patient SLC22A3^high^) showed that higher H3K4me3 and H3K4me3Q5Ser expression were observed in MES subtype tumor tissues, compared with adjacent non-cancerous (para-tumor) tissues (Normal SLC22A3^low^) (Fig. 6C).

H3K4-related transcription regulation was involved in various process in cancer development, including apoptosis, proliferation and ferroptosis. To further investigate the effect of serotonylation in transcriptional level, we analyzed the H3K4me3Q5Ser antibody binding DNA by CUT&Tag asssay. To examine the signal distribution of H3K4me3, CUT&Tag signals within a 3 kb region upstream and downstream of the Transcription Start Site (TSS) were statistically analyzed. SLC22A3 knockdown decreased the H3K4me3, expression on TSS, compared with NG (Fig. 6D). SLC22A3 knockdown resulted in the downregulation of a cluster of genes, which were enriched in metabolic and tumor-associated pathways according to KEGG analysis (Fig. 6E). The down-regulation of mRNA in RNA-sequencing results and H3K4me3Q5Ser binding to DNA in CUT&Tag results following SLC22A3 knockdown were demonstrated by intersection analysis (Fig. 6F). KEGG analysis revealed that the overlapping genes were predominantly enriched in cancer pathway, metabolic pathway, and serotonergic synapse (Fig. 6G). We found that the promoter region in the DNA sequence of SIRT1 was binding with H3K4me3Q5Ser. CHIP assay showed that both H3K4me3 and H3K4me3Q5Ser antibody binding with promoter region of SIRT1(Fig. 6H). The protein and mRNA level of *sirt1* was decreased in SLC22A3 knockdown cells, compared with the controls (Fig. 6I, J). Timer 2.0 platform analysis revealed that the expression of SLC22A3 and sirt1 is positively correlated (Fig. 6K).

H3K4me3 plays a unique role in directly promoting RNAPII (RNA polymerase II) transcription initiation and regulating the RNAPII promoter-proximal release-pause process. Previous data suggest that it enhances the formation of the gene transcription initiation complex by recruiting proteins containing plant homeodomain (PHD) fingers, such as TATA-box binding protein-associated factor 3 (TAF3) and Chromodomain Helicase DNA Binding Protein 1 (CHD1) [33, 34]. Therefore, we investigated the impact of SLC22A3 on methylation levels and examined whether it modulates gene regulatory functions mediated by H3K4. Co-Immunoprecipitation analysis showed that the deletion of SLC22A3 inhibited the binding of methyltransferase WD Repeat Domain 5 (WDR5) to H3K4. Meanwhile, the binding of TAF3 and CHD1 were also inhibited in SLC22A3 knockdown cells (Fig. 6L).

These results suggest that silencing SLC22A3 reduces serotonylation and methylation levels, which disrupts the binding of methyltransferase and RNAPII, ultimately leading to a decrease in SIRT1 gene expression.

### SIRT1-FOXO1 axis was responsible for MAOA transcription

**Fig. 7:**
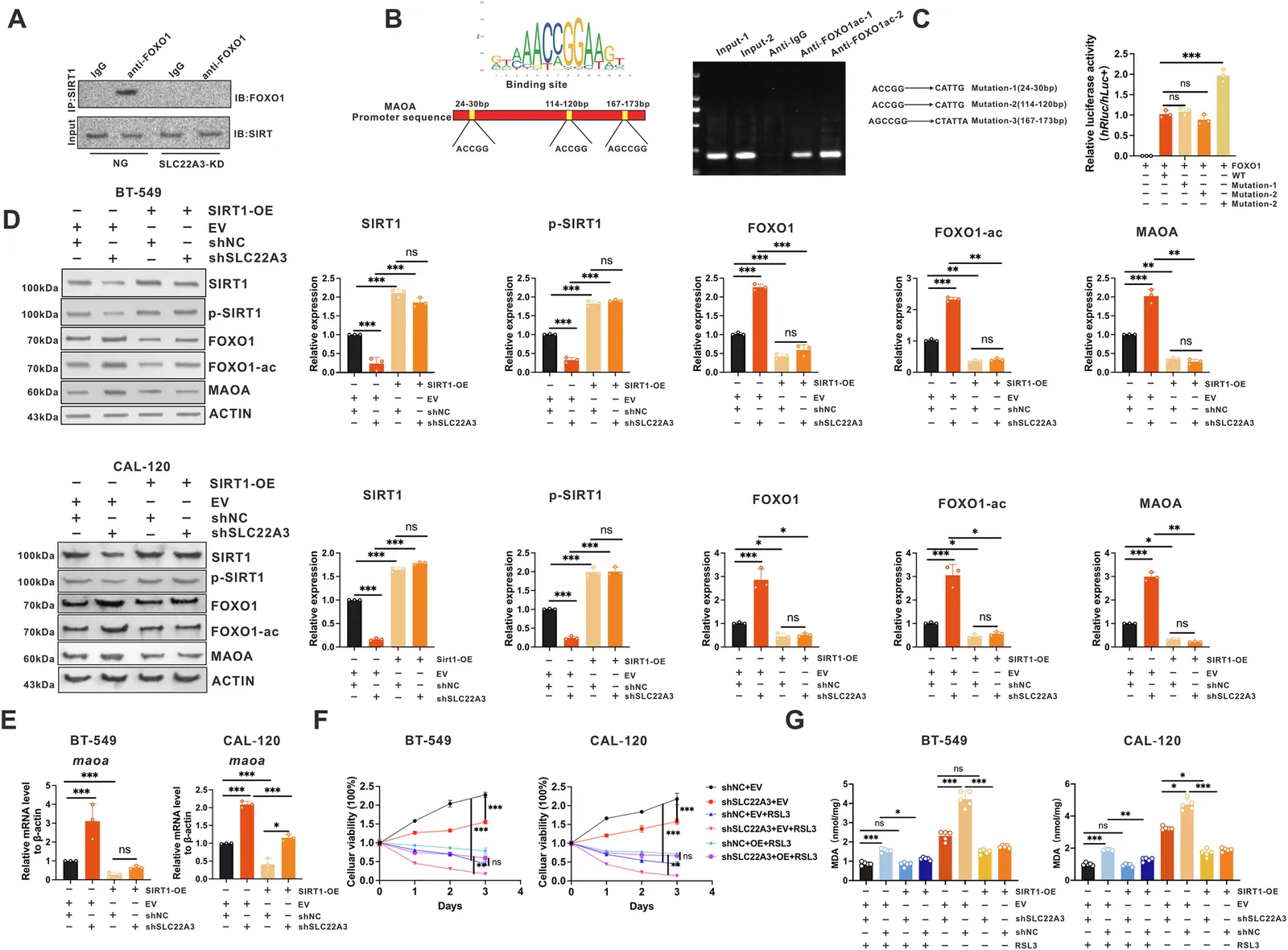
SIRT1-FOXO1 axis was responsible for MAOA transcription. **A** Co-inmunoprecipitation analyzed the interaction of FOXO1 with SIRT1. **B** Prediction binding sites of FOXO1 in MAOA promoter sequence by JASPAR, followed by Chip-PCR analysis. **C** Construction of luciferase reporter vectors (MAOA full-length promoter), and MAOA promoter binding site mutant. Luciferase activity assays were used to examine the functional binding site in MAOA affected by FOXO1. **D**, **E** The MES cells (BT-549 and CAL-120) were knockdown of or overexpression of SIRT1, followed with the expression of SIRT1, SIRT1 (phospho S47), FOXO1, Acetyl-FOXO1 (Lys294) and MAOA was determined by western blot (**D**). And the maoa mRNA expression was analyzed by RT-PCR (**E**) (*n* = 3). **F**, **G** The MES cells (BT-549 and CAL-120) were knockdown of or overexpression of SIRT1, followed with RSL3(10 μM) treatment for 8 h. The cell viability and MDA level of each group was detected by CCK8 assay (**F**) (*n* = 3), and ELISA (**G**), respectively (*n* = 5). Data are presented as mean ± SEM. *P < 0.05, **P < 0.01, ***P < 0.001 by 1-way ANOVA with Tukey’s posthoc test (**G** and **H**) or 2-way ANOVA (**C**–**G**).

SIRT1-FOXO1 signaling cascade participated in the regulation of cardiomyocyte ferroptosis and oxidative stress in previous reports [35]. Moreover, SIRT1 was down-regulated, which led to an increase in FOXO1 acetylation, which subsequently increased the transcription of MAOA in neuron cells [36]. In the current study, co-Immunoprecipitation verified the combination of FOXO1 and SIRT1 in BT-549 cells, which was depressed in SLC22A3-deficien cells (Fig. 7A). Bioinformatics analysis predicted three binding sites in the promoter of MAOA sequence and we identified the interaction of FOXO1 with MAOA promoter sequence by Chip-PCR (Fig. 7B). Further, we analyzed the potential binding site of FOXO1 on MAOA promoter sequence.

To further investigate it, a dual-luciferase reporter assay was constructed. As per the outcome of this analysis, FOXO1 regulated MAOA expression through binding with promoter of MAOA (167–173 kb) (Fig. 7C). Then, we verified the effect of sirt1 in regulation of MAOA expression through SIRT1-FOXO1. The results showed that SLC22A3 knockdown suppressed SIRT1 expression, promoted FOXO1 and acetylation expression, and enhanced MAOA expression. The expression of FOXO1 and MAOA was inhibited when SIRT1 was overexpressed in MES cells (BT-549 and CAL-120) (Fig. 7D, E). Next, SIRT1 was overexpressed in MES cells which enhanced the resistance to RSL3 (Fig. 7F and Supplementary Fig. S2) and greatly inhibited the MDA level in NG and SLC22A3 knockdown cells (Fig. 7G). These results indicated that SLC22A3 influence MAOA expression through regulating SIRT1-FOXO1 axis.

### TKIs targeted SLC22A3 renders cells sensitive to ferroptosis

**Fig. 8:**
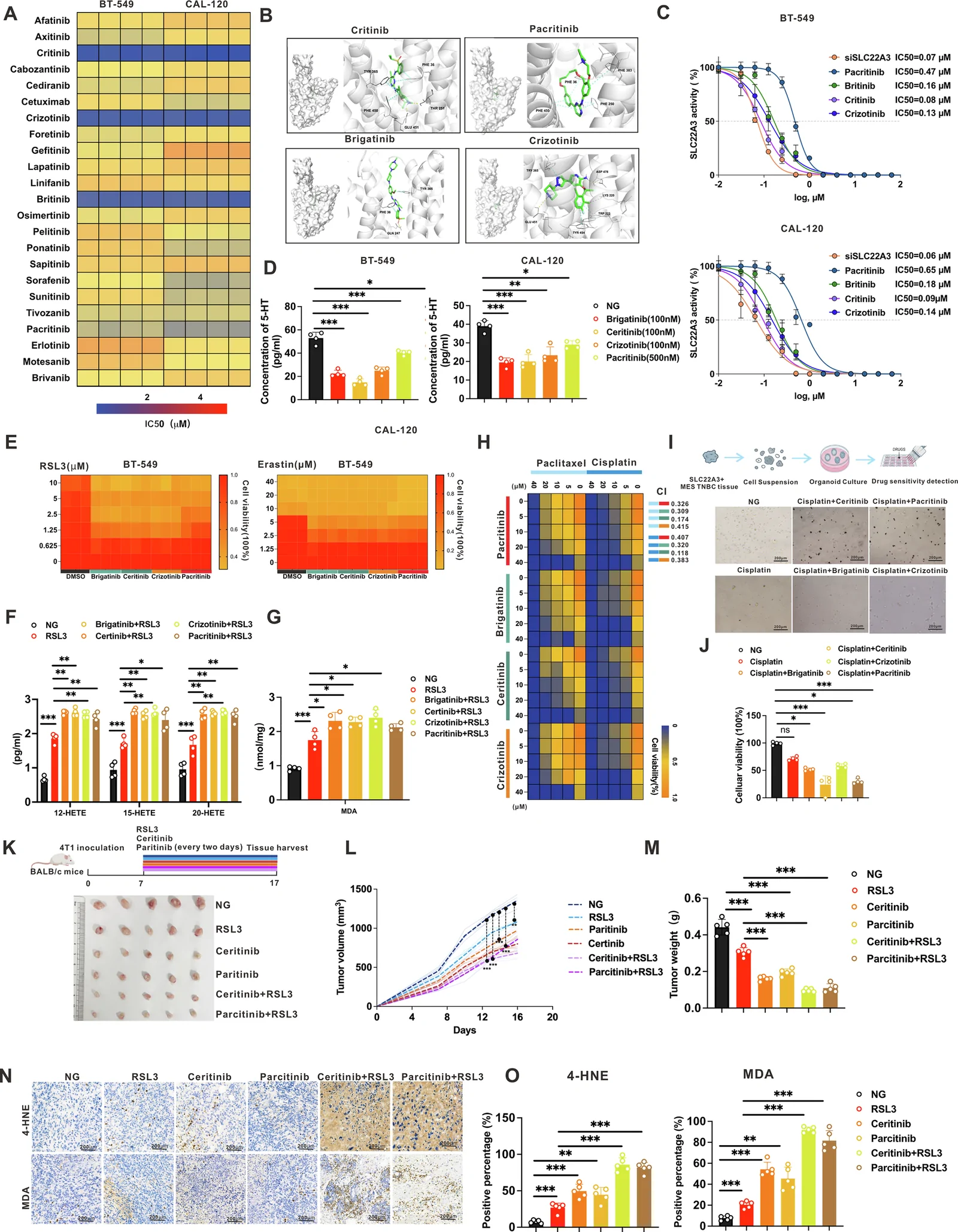
Sensitivity of ferroptosis was increased under TKIs treatment. **A** The inhibitory effects of 23 TKIs on SLC22A3 were determined by CCK8 assay. **B** Molecular docking of TKIs (Pacritinib, Britinib, Critinib and Crizotinib) with SLC22A3 protein (Blue:π-π interactions, Yellow: hydrogen bond interactions, Pink: salt bridge formed between the chlorine atom and the LYS 220 residue). **C** The SLC22A3 activity was measured under Pacritinib, Britinib, Critinib and Crizotinib treatment for different doses, as described in method “SLC22A3 activity measurement”. **D**, **E** The inhibitory effect of Critinib, Pacritinib, Cisplatin, Brigatinib and Crizotinib (500 nm) on MES subtypes tissue-derived organoids, and the cell viablity was analyzed by CCK8 assay (*n* = 4). **F** The concentration of 5-HT in MES cells (BT-549 and CAL-120) was measured by ELISA after Pacritinib, Britinib, Critinib and Crizotinib (100 nM) treatment for 8 h. **G** The sensitivity of MES cells (BT-549 and CAL-120) to RSL3 and Erasin was detected by CCK8. The HETEs (**H**) and MDA (**I**) of MES cells (BT-549 and CAL-120) were determined by ELISA after RSL3(10 μM) treatment. **J** BT-549 cells were subjected to single or combined treatments with TKIs (Pacritinib, Britinib, Critinib and Crizotinib), cisplatin, and paclitaxel for 12 h, followed by cell viability detection via the CCK-8 assay. The combination index (CI) was calculated according Chou-Talalay Method. **K**–**M** 4T1 cells were engrafted into mammary fat pads of BALB/c mice, followed with RSL3 (40 mg/kg) treatment, Pacritinib (5 mg/kg), or Critinib (5 mg/kg) every two days from day 7. The Tumor size (**K**) and tumor volume (**L**) in each group were measured. **N**, **O** The expression of MDA and 4NHE was measured in tumor tissues from different groups. The quantification was analyzed from five views (**O**). Data are presented as mean ± SEM. *P < 0.05, **P < 0.01, ***P < 0.001 by independent sample *t* test (**A**), 1-way ANOVA with Tukey’s posthoc test (**E**–**M**).

We aim to screen for SLC22A3-targeting drugs from common clinical chemotherapy agents for MES subtype patients. Tyrosine kinase inhibitors (TKIs) target receptor tyrosine kinases (RTKs) like VEGFR and EGFR, are mainly used for epithelial-related tumors, including NSCLC. We validated the inhibitory effects of 23 TKIs on SLC22A3 (Supplementary Data 1 and Fig. 8A). Four chemicals (Pacritinib, Britinib, Critinib and Crizotinib) exerted with robust inhibitory on SLC22A3. In addition, molecular dockings indicated that four drugs were significantly binding with protein SLC22A3, forming strong chemical bonds in active pocket, through π-π interactions, hydrogen bond interactions, salt bridge fromation between the chlorine atom and the LYS 220 residue (Fig. 8B). We verified that the TKIs (Pacritinib, Britinib, Critinib and Crizotinib) inhibited the activity of protein SLC22A3, with IC50 values of 0.08–0.65 μM, respectively (Fig. 8C). We treated the MES-TNBC-derived organoids with different TKIs and cisplatin. Cell viability assays revealed divergent responses to the treatments. Pacritinib and Critinib showed robust inhibition on MES-TNBC tissue-derived organoids, compared with controls and cisplatin (Fig. 8D, E). Meanwhile, they also decreased the intracellular concentration of 5-HT, increased the sensitivity of BT-549 cell to RSL3 and Erasin (Fig. 8F, G), as well as the productions of HETEs and MDA under RSL3 stimulation (Fig. 8H, I).

Further, the synergistic effects of combining TKIs with cisplatin or paclitaxel were investigated in vitro. Our screening identified TKIs that synergize with cisplatin and paclitaxel, thereby effectively inhibited MES cell viability (Fig. 8J). We validated the anticancer therapeutic effects of Pacritinib and Critinib with combination of RSL3 in vivo. 4T1 cells were injected to generate a syngenetic BALB/c mouse model. Mice were subdivided into six groups and respectively administered with RSL3, Ceritinib, Paritinib, RSL3 plus Ceritinib, and RSL3 plus Paritinib. The combination treatments significantly inhibited tumor growth and reduced tumor weight compared to RSL3 alone (Fig. 8K–M). Additionally, a significant increase in MDA and 4-HNE expression was observed in the tumor tissues of the co-treatment groups compared to those with the single treatment group (Fig. 8N, O). These results suggested that the Pacritinib and Critinib could be potential drugs in the treatment MES subtype patients.

## Discussion

The high heterogeneity of triple-negative breast cancer (TNBC) is a major limiting factor constraining its clinical treatment efficacy. Currently, no specific diagnostic or therapeutic guidelines have been established for TNBC [37, 38]. Traditionally defined solely by the absence of ER, PR, and HER2 expression, this definition masks significant internal variations—including genetic mutations, signaling pathway activation, and immune microenvironment differences—resulting in substantial variations in patient responses to existing standard treatments (primarily chemotherapy followed by radiation therapy). Consequently, recent research has increasingly focused on molecular subtyping of TNBC. An in-depth understanding of driver genes, signaling pathways, immune characteristics (such as PD-L1 expression and tumor-infiltrating lymphocytes [TILs]), and metabolic features across different molecular subtypes will facilitate the discovery of novel, subtype-specific therapeutic targets, enabling the development of personalized treatment strategies for individual patients [4, 39]. Recently, Shao et al. classified TNBC into four subtypes: luminal androgen receptor (LAR), immunomodulatory (IM), basal-like immune-suppressed (BLIS), and mesenchymal-like (MES). They also displayed ferroptosis heterogeneity, with the LAR subtype exhibiting high sensitivity to ferroptosis inducers, while the MES subtype remained resistant [6, 15]. In this study, we elucidated the mechanisms underlying ferroptosis resistance in MES-subtype TNBC and suggest SLC22A3 as a potential therapeutic target for treating this specific subtype.

First, by analyzing samples of the four molecular subtypes (IM, LAR, BLIS, MES) from the TCGA database as previously reported in the literature, we found that SLC22A3 expression was significantly upregulated in MES-subtype compared to the other three subtypes. Immunohistochemistry (IHC) results demonstrated that SLC22A3 expression levels were significantly higher in tumor tissues of MES-subtype patients than in adjacent non-tumor tissues, with predominant expression in tumor epithelial cells. Furthermore, multi-subtype analysis using TIMER 2.0 and IHC validation in MES samples consistently confirmed a positive correlation between SLC22A3 expression and the expression of MES-subtype signature molecules JAK1, STAT3, and FOXP3. Therefore, SLC22A3 may represent a novel biomarker for MES- TNBC.

SLC22A3 is a crucial organic cation transporter mediating the transmembrane transport of various endogenous cations, including 5-hydroxytryptamine (5-HT, serotonin) [24]. Interestingly, we found that 5-HT levels were upregulated in MES subtype cells and showed a positive correlation with SLC22A3 expression. Furthermore, RNA-sequencing and KEGG pathway enrichment analysis revealed that in SLC22A3-knockout MES-subtype breast cancer cell lines, 5-HT levels were significantly decreased (even under conditions of exogenous 5-HT supplementation), while the expression of ferroptosis pathway-related genes was significantly upregulated. Previous research has reported that, in addition to mediating neuronal activity, as well as the proliferation and invasion of cancer cells, 5-HT acts as a radical-trapping antioxidant (RTA) to eliminate lipid peroxidation, thereby resisting ferroptosis [31]. Therefore, we speculated that high levels of SLC22A3 promoted intracellular 5-HT transport, thereby conferring a ferroptosis-resistant phenotype on the MES subtype. Therefore, we speculated that high levels of SLC22A3 promoted intracellular 5-HT transport, thereby conferring a ferroptosis-resistant phenotype on the MES subtype.

It has been reported that 5-HT participates in various cellular functions through HTR-mediated pathways [40–42]. However, our data indicated that 5-HT-induced ferroptosis resistance is dependent on SLC22A3 but independent of HTRs. The fundamental cause of ferroptosis is the excessive lipid peroxidation of intracellular polyunsaturated fatty acids (PUFAs) in the presence of iron, which ultimately destroys the cell membrane structure and leads to cell death [8, 11, 43]. The general process can be summarized as follows: (1) Iron ions initiate lipid peroxidation through the Fenton reaction [44]; (2) PUFAs, as substrates, undergo chain reactions to generate a large number of oxidation products (such as OxPE and MDA) [9]; (3) The GPX4-GSH system and FSP1-CoQ perform antioxidant functions, and damage to either system may trigger ferroptosis [45]; (4) Lipid metabolizing enzymes such as LOXs catalyze the production of lipid peroxides, accelerating ferroptosis [46]. We detected key molecules involved in ferroptosis, and the results showed that compared with other subtypes (non-TNBC, BLIS, and LAR), the MES cell lines (BT-549 and CAL-120) exhibited stronger resistance to ferroptosis inducers (RSL3 and Erastin), and supplementation with 5-HT exacerbated this resistance. Conversely, cells with knockout of SLC22A3 showed increased sensitivity to ferroptosis, accompanied by elevated levels of ROS, PUFAs, LOXs (5-LOX, 8-LOX, 12-LOX, 15-LOX), free fatty acid peroxides HETEs, phospholipid peroxides OxPE, and malondialdehyde (MDA). However, 5-HT addition could not counteract these effects. We also observed that knockout of SLC22A3 did not affect the expression of SLC7A11 and GPX4. In vivo mouse tumor-bearing model showed that SLC22A3-knockout 4T1 and BT-549 cells both exhibited increased sensitivity to the ferroptosis inducer RSL3, suppressed tumor growth, and markedly elevated levels of MDA and 4-HNE in tumor tissues. Collectively, these in vitro and in vivo results demonstrated that SLC22A3 deficiency impaired 5-HT transport, leading to accumulation of intracellular oxidative metabolites and consequently enhancing ferroptosis susceptibility in MES cells.

In addition to its role as an antioxidant, 5-HT plays a critically important role in the epigenetic mechanisms of gene regulation. Catalyzed by transglutaminase 2 (TGM2), 5-HT modifies the fifth glutamine residue of histone H3 (H3Q5ser) [47]. This serotonylation modification triggered chromatin decondensation and drived the formation of neutrophil extracellular traps (NETs), promoting liver metastasis in prostate cancer. Importantly, TGM2 can serotonylate histone H3 on nucleosomes marked by trimethylated lysine 4 (H3K4me3), resulting in the combinatorial modification H3K4me3Q5ser. The Q5ser modification alters the interaction of specific proteins, particularly transcription initiation factors, with H3K4me3, thereby activating the transcription of target genes. We observed significantly downregulated levels of both H3K4me3 and H3K4me3Q5ser modifications in SLC22A3-knockout MES cells. By bioinformatics analysis, RNA sequencing, and ChIP experiments, we confirmed that the promoter region of *sirt1* contains binding sites for both H3K4me3 and H3K4me3Q5ser. Mechanistic studies revealed that SLC22A3 knockout markedly inhibited the recognition of H3K4 by the methyltransferase WD Repeat Domain 5 (WDR5), leading to reduced H3K4me3 levels. Concurrently, SLC22A3 knockout also impaired the recognition of H3K4me3 by transcription initiation factors such as CHD1 and TAF3, preventing the formation of the RNA polymerase II preinitiation complex (RNAPII PIC) and consequently suppressing the initiation of sirt1 gene transcription. Our results proved that both mRNA and protein levels of SIRT1 are significantly downregulated in SLC22A3-knockdown cells. Consistence with these findings, decreased expression of H3K4, H3K4me3Q5ser and SIRT1 were observed after MES cells treated with TGM2 inhibitor, a key enzyme-mediated histone serotonylation, as well as decreased resistance to RSL3 and Erasin, compared with the controls (Supplementary Fig. S2I, J). The SIRT1-FOXO1-MAOA signaling pathway has been reported to regulate ferroptosis in cardiomyocytes. MAOA is a key enzyme in monoamine metabolism, primarily responsible for inactivating monoamine neurotransmitters. High expression of monoamine oxidase promotes the catabolism of 5-HT into 5-hydroxyindole-acetic acid (5-HIAA), generating H_2_O_2_ in the process, which causes cellular damage. We further demonstrated that SLC22A3 knockout led to down-regulated SIRT1 expression, elevated levels of acetylated FOXO1, and upregulated expression of monoamine oxidase MAOA, ultimately increasing ferroptosis sensitivity in MES cells.

Combination chemotherapy regimens containing paclitaxel, cyclophosphamide, cisplatin, and fluorouracil are recommended for TNBC patients. Traditional chemotherapy may be ineffective and lead to long-term complications, impacting the patient’s quality of life. The FUTURE trial, involving 69 patients with multi-drug-resistant, metastatic triple-negative breast cancer, showed a less effective treatment outcome for the MES subtype compared to other subtypes, highlighting the need for improved strategies for this patient group [26]. In this study, we screened clinically approved TKIs and identified pacritinib, brigatinib, ceritinib, and crizotinib as potent inhibitors of SLC22A3. These TKIs exerted therapeutic effects by inhibiting 5-HT uptake, thereby suppressing tumor growth and inducing ferroptosis. They synergized with cisplatin and paclitaxel to enhance antitumor activity. Of particular interest, ceritinib and pacritinib effectively suppressed the growth of MES-TNBC-derived organoids, highlighting their potential as promising therapeutic candidates for MES-TNBC.

The limitation of this study lies in the fact that, as there are currently no suitable animal models that fully replicates the mesenchymal (MES) subtype of triple-negative breast cancer, we employed orthotopic inoculation of 4T1 cells into the mammary fat pad of BALB/c mice. This model exhibits certain differences from the characteristics of the MES subtype. For future in-depth research, organoid models derived from tumor tissues of MES subtype breast cancer patients could be considered for relevant evaluations. Moreover, although the SLC22A3 is mainly expressed in cancer epithelial cell and CAF, the function of SLC22A3 was not investigated in CAF cells in the current study.

## Conclusion

This study elucidated the mechanism underlying ferroptosis resistance in the MES subtype of triple-negative breast cancer. The MES subtype exhibited high expression of SLC22A3, which regulated the uptake and metabolism of serotonin (5-HT), thereby influencing tumor cell sensitivity to ferroptosis. Intracellular 5-HT primarily functioned through two pathways: Firstly, acting as a radical-trapping antioxidant (RTA) to scavenge lipid peroxides and inhibit ferroptosis. Secondly, inducing histone serotonylation, which not only enhanced histone methylation levels but also promoted the recognition of methylated histones by transcription initiation factors, facilitating the assembly of the transcription initiation complex. This ultimately led to transcriptional activation of the downstream *sirt1* gene. SIRT1 inhibited the acetylation of FOXO1, suppressing MAOA transcription and reducing 5-HT degradation, thereby promoting ferroptosis resistance. Our findings indicate that targeting SLC22A3, in combination with ferroptosis inducers, represents a potential therapeutic strategy for patients with MES-subtype breast cancer.

## Supplementary information

Supplementary figure

Supplementary figure S1

Supplementary figure S2

Supplemntary data1

Supplemntary data2

Original western blot

## Data Availability

All data can be viewed in The National Omics Data Encyclopedia (http://www.biosino.org/node) by pasting the accession (PRJCA061458) into the text search box or through the URL: https://www.cncb.ac.cn/. Single cell RNA-sequencing data and metadata are available at GEO Datasets GSE176078. This paper does not report the original code. Any additional information required to reanalyze the data reported in this paper is available from the lead contact upon request.

## References

[1] Vagia E, Mahalingam D, Cristofanilli M. The LANDSCAPE OF TARGETED THERApies in TNBC. Cancers. 2020;12:916. 10.3390/cancers12040916 PMC7226210 32276534

[2] Fines C, McCarthy H, Buckley N. The search for a TNBC vaccine: the guardian vaccine. Cancer Biol Ther. 2025;26:2472432. 10.1080/15384047.2025.2472432 PMC11913391 40089851

[3] Lehmann BD, Pietenpol JA. Identification and use of biomarkers in treatment strategies for triple-negative breast cancer subtypes. J Pathol. 2014;232:142–50. 10.1002/path.4280 PMC4090031 24114677

[4] Lehmann BD, Colaprico A, Silva TC, Chen J, An H, Ban Y, et al. Multi-omics analysis identifies therapeutic vulnerabilities in triple-negative breast cancer subtypes. Nat Commun. 2021;12:6276. 10.1038/s41467-021-26502-6 PMC8560912 34725325

[5] Burstein MD, Tsimelzon A, Poage GM, Covington KR, Contreras A, Fuqua SA, et al. Comprehensive genomic analysis identifies novel subtypes and targets of triple-negative breast cancer. Clin Cancer Res. 2015;21:1688–98. 10.1158/1078-0432.CCR-14-0432 PMC4362882 25208879

[6] Jiang YZ, Ma D, Suo C, Shi J, Xue M, Hu X, et al. Genomic and transcriptomic landscape of triple-negative breast cancers: subtypes and treatment strategies. Cancer cell. 2019;35:428–40.e5. 10.1016/j.ccell.2019.02.001 30853353

[7] Zhao S, Ma D, Xiao Y, Li XM, Ma JL, Zhang H, et al. Molecular subtyping of triple-negative breast cancers by immunohistochemistry: molecular basis and clinical relevance. oncologist. 2020;25:e1481–e91. 10.1634/theoncologist.2019-0982 PMC7543239 32406563

[8] Dixon SJ, Lemberg KM, Lamprecht MR, Skouta R, Zaitsev EM, Gleason CE, et al. Ferroptosis: an iron-dependent form of nonapoptotic cell death. Cell. 2012;149:1060–72. 10.1016/j.cell.2012.03.042 PMC3367386 22632970

[9] Conrad M, Pratt DA. The chemical basis of ferroptosis. Nat Chem Biol. 2019;15:1137–47. 10.1038/s41589-019-0408-1 31740834

[10] Dixon SJ, Olzmann JA. The cell biology of ferroptosis. Nat Rev Mol cell Biol. 2024;25:424–42. 10.1038/s41580-024-00703-5 PMC12187608 38366038

[11] Kagan VE, Mao G, Qu F, Angeli JP, Doll S, Croix CS, et al. Oxidized arachidonic and adrenic PEs navigate cells to ferroptosis. Nat Chem Biol. 2017;13:81–90. 10.1038/nchembio.2238 PMC5506843 27842066

[12] Dixon SJ, Winter GE, Musavi LS, Lee ED, Snijder B, Rebsamen M, et al. Human haploid cell genetics reveals roles for lipid metabolism genes in nonapoptotic cell death. ACS Chem Biol. 2015;10:1604–9. 10.1021/acschembio.5b00245 PMC4509420 25965523

[13] Su Y, Zhao B, Zhou L, Zhang Z, Shen Y, Lv H, et al. Ferroptosis, a novel pharmacological mechanism of anti-cancer drugs. Cancer Lett. 2020;483:127–36. 10.1016/j.canlet.2020.02.015 32067993

[14] Soula M, Weber RA, Zilka O, Alwaseem H, La K, Yen F, et al. Metabolic determinants of cancer cell sensitivity to canonical ferroptosis inducers. Nat Chem Biol. 2020;16:1351–60. 10.1038/s41589-020-0613-y PMC8299533 32778843

[15] Yang F, Xiao Y, Ding JH, Jin X, Ma D, Li DQ, et al. Ferroptosis heterogeneity in triple-negative breast cancer reveals an innovative immunotherapy combination strategy. Cell Metab. 2023;35:84–100.e8. 10.1016/j.cmet.2022.09.021 36257316

[16] Nguyen TA, Le MK, Nguyen PT, Tran NQV, Kondo T, Nakao A. SLC22A3 that encodes organic cation transporter-3 is associated with prognosis and immunogenicity of human lung squamous cell carcinoma. Transl Lung Cancer Res. 2023;12:1972–86. 10.21037/tlcr-23-334 PMC10654437 38025816

[17] Sardar D, Cheng YT, Woo J, Choi DJ, Lee ZF, Kwon W, et al. Induction of astrocytic Slc22a3 regulates sensory processing through histone serotonylation. Science. 2023;380:eade0027. 10.1126/science.ade0027 PMC10874521 37319217

[18] Ling T, Dai Z, Wang H, Kien TT, Cui R, Yu T, et al. Serotonylation in tumor-associated fibroblasts contributes to the tumor-promoting roles of serotonin in colorectal cancer. Cancer Lett. 2024;600:217150. 10.1016/j.canlet.2024.217150 39097134

[19] Seo E, Jee B, Chung JH, Song W, Sung HH, Jeon HG, et al. Repression of SLC22A3 by the AR-V7/YAP1/TAZ axis in enzalutamide-resistant castration-resistant prostate cancer. FEBS J. 2023;290:1645–62. 10.1111/febs.16657 36254631

[20] Gu Y, Xu ZJ, Zhou JD, Wen XM, Jin Y, Yuan Q, et al. SLC22A3 methylation-mediated gene silencing predicts adverse prognosis in acute myeloid leukemia. Clin Epigenet. 2022;14:162. 10.1186/s13148-022-01373-w PMC9716704 36461046

[21] Cervenkova L, Vycital O, Bruha J, Rosendorf J, Palek R, Liska V, et al. Protein expression of ABCC2 and SLC22A3 associates with prognosis of pancreatic adenocarcinoma. Sci Rep. 2019;9:19782. 10.1038/s41598-019-56059-w PMC6930301 31874997

[22] Zhu W, Liu X, Yang L, He Q, Huang D, Tan X. Ferroptosis and tumor immunity: In perspective of the major cell components in the tumor microenvironment. Eur J Pharmacol. 2023;961:176124. 10.1016/j.ejphar.2023.176124 37925133

[23] Liu C, Li J, Xu F, Chen L, Ni M, Wu J, et al. PARP1-DOT1L transcription axis drives acquired resistance to PARP inhibitor in ovarian cancer. Mol cancer. 2024;23:111. 10.1186/s12943-024-02025-8 PMC11110363 38778348

[24] Alim K, Moreau A, Bruyère A, Jouan E, Denizot C, Nies AT, et al. Inhibition of organic cation transporter 3 activity by tyrosine kinase inhibitors. Fundam Clin Pharmacol. 2021;35:919–29. 10.1111/fcp.12657 33523504

[25] Sayyed K, Camillerapp C, Le Vée M, Bruyère A, Nies AT, Abdel-Razzak Z, et al. Inhibition of organic cation transporter (OCT) activities by carcinogenic heterocyclic aromatic amines. Toxicol Vitr. 2019;54:10–22. 10.1016/j.tiv.2018.08.015 30189235

[26] Liu Y, Zhu XZ, Xiao Y, Wu SY, Zuo WJ, Yu Q, et al. Subtyping-based platform guides precision medicine for heavily pretreated metastatic triple-negative breast cancer: The FUTURE phase II umbrella clinical trial. Cell Res. 2023;33:389–402. 10.1038/s41422-023-00795-2 PMC10156707 36973538

[27] Bradford MM. A rapid and sensitive method for the quantitation of microgram quantities of protein utilizing the principle of protein-dye binding. Anal Biochem. 1976;72:248–54. 10.1016/0003-2697(76)90527-3 942051

[28] Tate CR, Rhodes LV, Segar HC, Driver JL, Pounder FN, Burow ME, et al. Targeting triple-negative breast cancer cells with the histone deacetylase inhibitor panobinostat. Breast cancer Res : BCR. 2012;14:R79. 10.1186/bcr3192 PMC3446342 22613095

[29] Rhodes LV, Muir SE, Elliott S, Guillot LM, Antoon JW, Penfornis P, et al. Adult human mesenchymal stem cells enhance breast tumorigenesis and promote hormone independence. Breast Cancer Res Treat. 2010;121:293–300. 10.1007/s10549-009-0458-2 PMC4943323 19597705

[30] Atadja P. Development of the pan-DAC inhibitor panobinostat (LBH589): successes and challenges. Cancer Lett. 2009;280:233–41. 10.1016/j.canlet.2009.02.019 19344997

[31] Liu D, Liang CH, Huang B, Zhuang X, Cui W, Yang L, et al. Tryptophan metabolism acts as a new anti-ferroptotic pathway to mediate tumor growth. Adv Sci. 2023;10:e2204006. 10.1002/advs.202204006 PMC9951368 36627132

[32] Tu RH, Wu SZ, Huang ZN, Zhong Q, Ye YH, Zheng CH, et al. Neurotransmitter receptor HTR2B regulates lipid metabolism to inhibit ferroptosis in gastric cancer. Cancer Res. 2023;83:3868–85. 10.1158/0008-5472.CAN-23-1012 38037454

[33] Farrelly LA, Thompson RE, Zhao S, Lepack AE, Lyu Y, Bhanu NV, et al. Histone serotonylation is a permissive modification that enhances TFIID binding to H3K4me3. Nature. 2019;567:535–9. 10.1038/s41586-019-1024-7 PMC6557285 30867594

[34] Wang H, Fan Z, Shliaha PV, Miele M, Hendrickson RC, Jiang X, et al. H3K4me3 regulates RNA polymerase II promoter-proximal pause-release. Nature. 2023;615:339–48. 10.1038/s41586-023-05780-8 PMC9995272 36859550

[35] Ju J, Li XM, Zhao XM, Li FH, Wang SC, Wang K, et al. Circular RNA FEACR inhibits ferroptosis and alleviates myocardial ischemia/reperfusion injury by interacting with NAMPT. J Biomed Sci. 2023;30:45. 10.1186/s12929-023-00927-1 PMC10304620 37370086

[36] Li Y, Jiao Q, Du X, Jiang H. Sirt1/FoxO1-associated MAO-A upregulation promotes depressive-like behavior in transgenic mice expressing human A53T α-synuclein. ACS Chem Neurosci. 2020;11:3838–48. 10.1021/acschemneuro.0c00628 33155799

[37] Zagami P, Carey LA. Triple negative breast cancer: pitfalls and progress. NPJ breast cancer. 2022;8:95. 10.1038/s41523-022-00468-0 PMC9392735 35987766

[38] von Minckwitz G, Martin M. Neoadjuvant treatments for triple-negative breast cancer (TNBC). Ann Oncol. 2012;23:vi35–9. 10.1093/annonc/mds193 23012300

[39] Gong Y, Ji P, Yang YS, Xie S, Yu TJ, Xiao Y, et al. Metabolic-pathway-based subtyping of triple-negative breast cancer reveals potential therapeutic targets. Cell Metab. 2021;33:51–64.e9. 10.1016/j.cmet.2020.10.012 33181091

[40] Karmakar S, Lal G. Role of serotonin receptor signaling in cancer cells and anti-tumor immunity. Theranostics. 2021;11:5296–312. 10.7150/thno.55986 PMC8039959 33859748

[41] León-Ponte M, Ahern GP, O’Connell PJ. Serotonin provides an accessory signal to enhance T-cell activation by signaling through the 5-HT7 receptor. Blood. 2007;109:3139–46. 10.1182/blood-2006-10-052787 PMC1852236 17158224

[42] Curtis JJ, Vo NTK, Seymour CB, Mothersill CE. 5-HT(2A) and 5-HT(3) receptors contribute to the exacerbation of targeted and non-targeted effects of ionizing radiation-induced cell death in human colon carcinoma cells. Int J Radiat Biol. 2020;96:482–90. 10.1080/09553002.2020.1704911 31846381

[43] Kagan VE, Tyurina YY, Vlasova II, Kapralov AA, Amoscato AA, Anthonymuthu TS, et al. Redox epiphospholipidome in programmed cell death signaling: catalytic mechanisms and regulation. Front Endocrinol. 2020;11:628079. 10.3389/fendo.2020.628079 PMC7933662 33679610

[44] Minotti G, Aust SD. The role of iron in oxygen radical mediated lipid peroxidation. Chem Biol Interact. 1989;71:1–19. 10.1016/0009-2797(89)90087-2 2550151

[45] Bersuker K, Hendricks JM, Li Z, Magtanong L, Ford B, Tang PH, et al. The CoQ oxidoreductase FSP1 acts parallel to GPX4 to inhibit ferroptosis. Nature. 2019;575:688–92. 10.1038/s41586-019-1705-2 PMC6883167 31634900

[46] Magtanong L, Ko PJ, To M, Cao JY, Forcina GC, Tarangelo A, et al. Exogenous monounsaturated fatty acids promote a ferroptosis-resistant cell state. Cell Chem Biol. 2019;26:420–32.e9. 10.1016/j.chembiol.2018.11.016 PMC6430697 30686757

[47] Dong R, Wang T, Dong W, Zhang H, Li Y, Tao R, et al. TGM2-mediated histone serotonylation promotes HCC progression via MYC signalling pathway. J Hepatol. 2025;83:105–18. 10.1016/j.jhep.2024.12.038 39788430

