# Supplementary material for: SLC22A3 regulates ferroptosis in the mesenchymal subtype of triple-negative breast cancer by modulating histone H3K4 serotonylation
Source: Cell Death Dis. 2026 Jul 9;17(1):855. doi: 10.1038/s41419-026-09057-6 (PMC13633284; doi:10.1038/s41419-026-09057-6)
Supplement: Supplementary file 1 [file 41419_2026_9057_MOESM1_ESM.docx]

**Supplementary S1**

(A) The mRNA expression of SLC22A3 in MES (CAL-51,CAL-120,HCC1395,HCC38,BT-549) and non-MES cells (HCC1937,HCC1806,MDAMB231,MDAMB453,BT20).
(B) The expression of SLC22A3 cells in different cell types (MCF-7, HCC1937, MDAMB453, BT-549, CAL-120) (n=4).
(A-D) The SLC22A3 was knockdown by lenti-virus in BT-549 and CAL-120 cells, and the expression of SLC22A3 was determined by western blot (n=4). The β-actin was used as internal control

(E)The SLC22A3 was knockdown by lenti-virus in BT-549 and CAL-120 cells, and the expression of SLC22A1 and SLC22A2 was determined by western blot (n=4). The β-actin was used as internal control.

(F) The concentration of cellular 5-HT in control (Negative control, NG) and SLC22A3 knockdown (SLC22A3-KD) cells was determined by ELISA(n=4).

(G)The MES cells (BT-549 and CAL-120) were treated with different doses of 5-HT and the sensitivity to RSL3 was detected by CCK8(n=4).

(H) The SLC22A3 was over-expressed in HCC1937 and MDAMB453, and the expression of SLC22A3 was determined by western blot (n=4). The β-actin was used as internal control.

(I-J) The sensitivity of Negative controls (NG) and SLC22A3 overexpression HCC1937 and MDAMBB453 cells to inducer of ferroptosis (RSL3 and Erasin), n=3. Data are presented as mean ± SEM. **P* < 0.05, ***P* < 0.01, ****P* < 0.001 by independent sample t test (A,B,H) or 1-way ANOVA with Tukey’s posthoc test (B-F) .

**Supplementary S2**

(A-B) The MAOA was knockdown or over-expressed in MES cells (BT-549 and CAL-120), and the expression of MAOA was determined by western blot (n=4).

(C) The SIRT1 was over-expressed in MES cells (BT-549 and CAL-120), and the expression of SIRT1 was determined by western blot(n=4).

(D) MES cells (BT-549 and CAL-120) were treated with TGM2 inhibitor (TG-2-IN-1,1μM) and different doses of RSL3 or Erasin respectively. The cell viability was detected by CCK8.

(E-F) The expression of SIRT1, p-SIRT1, H3K4me3, H3K4me3QSer was analyzed after MES cells were treated with TGM2 inhibitor (TG-2-IN-1,1μM) for 24 hours. The β-actin was used as internal control (e). The qualification of the western blot intensity (n=4) (f). Data are presented as mean ± SEM. **P* < 0.05, ***P* < 0.01, ****P* < 0.001 by independent sample t test (F) or 1-way ANOVA with Tukey’s posthoc test (A-B) .
