# Supplementary material for: SLC22A3 regulates ferroptosis in the mesenchymal subtype of triple-negative breast cancer by modulating histone H3K4 serotonylation
Source: Cell Death Dis. 2026 Jul 9;17(1):855. doi: 10.1038/s41419-026-09057-6 (PMC13633284; doi:10.1038/s41419-026-09057-6)
Supplement: Supplementary file 2 [file 41419_2026_9057_MOESM5_ESM.docx]

**Table 1**

Information of TNBC patients

| NO. | Number | Age | Gender | HER2 | PR | ER | CD8 | AR | FOXC1 | DCLK | Subtype |
| --- | --- | --- | --- | --- | --- | --- | --- | --- | --- | --- | --- |
| 1 | TD-20230326 | 51 | Female | **-** | **-** | **-** | **-** | **+** | **-** | **-** | LAR |
| 2 | TD-20230327 | 62 | Female | **-** | **-** | **-** | **-** | **+** | **-** | **-** | LAR |
| 3 | TD-20230402 | 48 | Female | **-** | **-** | **-** | **+** | **-** | **-** | **-** | IM |
| 4 | TD-20230405 | 59 | Female | **-** | **-** | **-** | **-** | **+** | **-** | **-** | LAR |
| 5 | TD-20230409 | 66 | Female | **-** | **-** | **-** | **+** | **-** | **-** | **-** | IM |
| 6 | TD-20230420 | 49 | Female | **-** | **-** | **-** | **+** | **-** | **-** | **-** | IM |
| 7 | TD-20230422 | 44 | Female | **-** | **-** | **-** | **-** | **+** | **-** | **-** | LAR |
| 8 | TD-20230519 | 66 | Female | **-** | **-** | **-** | **-** | **-** | **-** | **+** | MES |
| 9 | TD-20230623 | 71 | Female | **-** | **-** | **-** | **-** | **-** | **+** | **-** | BILS |
| 10 | TD-20230629 | 52 | Female | **-** | **-** | **-** | **-** | **-** | **+** | **-** | BILS |
| 11 | TD-20230709 | 56 | Female | **-** | **-** | **-** | **-** | **+** | **-** | **-** | LAR |
| 12 | TD-20230711 | 51 | Female | **-** | **-** | **-** | **-** | **+** | **-** | **-** | LAR |
| 13 | TD-20230716 | 74 | Female | **-** | **-** | **-** | **-** | **-** | **+** | **-** | BILS |
| 14 | TD-20230914 | 40 | Female | **-** | **-** | **-** | **-** | **+** | **-** | **-** | LAR |
| 15 | TD-20230920 | 66 | Female | **-** | **-** | **-** | **-** | **-** | **-** | **+** | MES |
| 16 | TD-20231009 | 65 | Female | **-** | **-** | **-** | **-** | **-** | **+** | **-** | BILS |
| 17 | TD-20231027 | 66 | Female | **-** | **-** | **-** | **-** | **-** | **+** | **-** | BILS |
| 18 | TD-20231030 | 63 | Female | **-** | **-** | **-** | **-** | **+** | **-** | **-** | LAR |
| 19 | TD-20231030-2 | 55 | Female | **-** | **-** | **-** | **+** | **-** | **-** | **-** | IM |
| 20 | TD-20231117 | 57 | Female | **-** | **-** | **-** | **+** | **-** | **-** | **-** | IM |
| 21 | TD-20231209 | 58 | Female | **-** | **-** | **-** | **-** | **-** | **-** | **+** | MES |
| 22 | TD-20231210 | 52 | Female | **-** | **-** | **-** | **+** | **-** | **-** | **-** | IM |
| 23 | TD-20231218 | 69 | Female | **-** | **-** | **-** | **-** | **+** | **-** | **-** | LAR |
| 24 | TD-20231229 | 49 | Female | **-** | **-** | **-** | **-** | **-** | **-** | **+** | MES |
| 25 | TD-20240304 | 62 | Female | **-** | **-** | **-** | **+** | **-** | **-** | **-** | IM |
| 26 | TD-20240311 | 67 | Female | **-** | **-** | **-** | **-** | **-** | **-** | **+** | MES |
| 27 | TD-20240322 | 77 | Female | **-** | **-** | **-** | **-** | **-** | **+** | **-** | BILS |
| 28 | TD-20240418 | 72 | Female | **-** | **-** | **-** | **-** | **-** | **+** | **-** | BILS |
| 29 | TD-20240418-2 | 51 | Female | **-** | **-** | **-** | **+** | **-** | **-** | **-** | IM |
| 30 | TD-20240518 | 59 | Female | **-** | **-** | **-** | **-** | **-** | **+** | **-** | BILS |
| 31 | TD-20240522 | 55 | Female | **-** | **-** | **-** | **-** | **-** | **+** | **-** | BILS |
| 32 | TD-20240704 | 53 | Female | **-** | **-** | **-** | **+** | **-** | **-** | **-** | IM |
| 33 | TD-20240719 | 52 | Female | **-** | **-** | **-** | **+** | **-** | **-** | **-** | IM |
| 34 | TD-20240830 | 48 | Female | **-** | **-** | **-** | **-** | **-** | **+** | **-** | BILS |
| 35 | TD-20241001 | 49 | Female | **-** | **-** | **-** | **+** | **-** | **-** | **-** | IM |

**Table 2**

| **Primer information** | | | |
| --- | --- | --- | --- |
| NO. | Gene name | Froward (5’-3’) | Reverse(5’-3’) |
| 1 | SLC22A3 | GCCCGGAGCTCTCTTAATCC | GCCACTATATTGCTTGCCGC |
| 2 | MAOA | GAATTCCTTCAGGGCCGGGCTCTTCC | TCCATCCGTACATTTTAAAGACT |
| 3 | MAOB | GAATTCTAGTGTGAGGGGACAGTAAGCAGC | ATCCAGTTTG CGAAATAACAAGACT |
| 4 | SIRT1 | TAGCCTTGTCAGATAAGGAAGGA | ACAGCTTCACAGTCAACTTTGT |
| 6 | SIRT1（Chip-PCR） | TAACATGGAAGATGGCGGACG | CCTCTGCCTCCGCTGTTC |
| 7 | MAOA (Promoter) | CCCCTTCC TCCGACTCCG | GCTTCGGCCCCCGTGTTGACGG |
| 8 | ACTIN | GGAGCGAGATCCCTCCAAAAT | GGCTGTTGTCATACTTCTCATGG |

**Table 3**

| **shRNA sequence information** | | |
| --- | --- | --- |
| NO. | Gene name | Target Sequence (5’-3’) |
| 1 | SLC22A3-1 | CCTAAGAGTGTTTCCCATGAT |
| 2 | SLC22A3-2 | CCTGAACCTCTCTTATTATAT |
| 3 | MAOA-1 | GCAGAGAGAAACCAGTTAATT |
| 3 | MAOA-2 | CGGATATTCTCTGTCACCAAT |
| 4 | SIRT1-1 | GCAAAGCCTTTCTGAATCTAT |
| 4 | SIRT1-2 | CCTCGAACAATTCTTAAAGAT |

**Table4**

**Antibodies and regents**

| **Antibodies** | | |
| --- | --- | --- |
| **Name** | **Company** | **Catalogue** |
| Anti-human SLC22A3 | ab124826 | Abcam |
| Anti-human ICAM1 | ab282575 | Abcam |
| Anti-human DAPI | C0065 | Abcam |
| Anti-human 5-HT | MERK | MAB352 |
| Anti-human JAK2 | Abcam | ab245303 |
| Anti-human STAT3 | Abcam | ab68153 |
| Anti-human FOXP3 | Abcam | ab20034 |
| Anti-CD4 | Elabscience | E-AB-F1097C |
| Anti-CD8 | Elabscience | E-AB-F1104D |
| Anti-CD25 | Elabscience | E-AB-F1102E |
| Anti-FOXP3 | Elabscience | E-AB-F1351J |
| Anti-IFN-ϒ | Elabscience | E-AB-F1101M1 |
| Anti-Histone H3Kme3Q5Ser | MERK | ABE2580 |
| Anti-H3K4me | abcam | ab176877 |
| Anti-H3K4me2 | abcam | ab32356 |
| Anti-H3K4me3 | abcam | ab213224 |
| Anti-Malondialdehyde | abcam | ab27642 |
| Anti-4 Hydroxynonenal | abcam | Ab46545 |
| Anti-H3K4me3 | abcam | ab213224 |
| Anti-MAOA | abcam | 10539-1-AP |
| Anti-MAOB | abcam | 12602-1-AP |
| Anti-TAF3 | abcam | ab188332 |
| Anti-WDR5 | abcam | ab307664 |
| Anti-CHD1 | abcam | ab318138 |
| Anti-SIRT1 | abcam | ab189494 |
| Anti- SIRT1 (phospho S47) | abcam | ab76039 |
| Anti-FOXO1A | abcam | ab39670 |
| Anti-FOXO1A | Affinity | AF2305 |
| Anti-Beta-ACTIN | proteintec | 60008-1-Ig |
| DyLight 594 | Proteintech |  |
| DAPI | Proteintech | 28718-90-3 |
| **Regents** | | |
| **Name** | **Company** | **Catalogue** |
| 1640 | Solarbio | 90023 |
| DMEM | Solarbio | 11965 |
| DMEM/F12 | Gibco | 65294 |
| Matrigel | Corning | CB-40234A |
| FBS | Solarbio | S9020 |
| RIPA | Solarbio | R0010 |
| 1% streptomycin/ penicillin | Biosharp | BL505A |
| DAB | Zsgb-bio | ZLI-9018 |
| Hematoxylin staining solution | Beyotime | C0107 |
| CCK8 | Beyotime | C0038 |
| 2×SYBR Green Pro Taq HS Premix | Accurate Biology | AG11702 |
| VAHTS DNA clean beads | VAHTS | #N411-03 |
| Cocktail | Solarbio | P1260 |
| ECL Substrate | Bio-Rad | 1705061 |
| C11-BODIPY 581/591 | Thermo Fisher Scientific | D3861 |
| RSL | MCE | HY-100218A |
| Erasin | MCE | HY-115594 |
| BODIPY™ 581/591 C11 | MCE | HY-D1301 |
| brigatinib | MCE | HY-12857 |
| crizotinib | MCE | HY-50878 |
| ceritinib | MCE | HY-15656 |
| pacritinib | MCE | HY-16379 |

**Kits**

| **Name** | **Company** | **Catalogue** |
| --- | --- | --- |
| 5-HETE ELISA Kit | Huamei biology | CSB-E17006h |
| 12-HETE ELISA Kit | abcam | ab133034 |
| 15-HETE ELISA Kit | abcam | ab133035 |
| 20-HETE ELISA Kit | abcam | ab175817 |
| 5-HT ELISA Kit | JiangLai biology | JL53170-48T |
| MDA ELISA Kit | solarbio | BC0020 |
| Wenfuxi | ShuWen biology | 20230512 |
| SimpleChIP® Enzymatic Chromatin IP Kit | CST | 9003 S |
| Evo M-MLV RT Master Mix Kit | Accurate Biology | AG11706 |
| BCA Protein Assay Kit | Beyotime | P0010 |
