# Supplementary material for: SLC22A3 regulates ferroptosis in the mesenchymal subtype of triple-negative breast cancer by modulating histone H3K4 serotonylation
Source: Cell Death Dis. 2026 Jul 9;17(1):855. doi: 10.1038/s41419-026-09057-6 (PMC13633284; doi:10.1038/s41419-026-09057-6)
Supplement: Supplementary file 3 [file 41419_2026_9057_MOESM6_ESM.docx]

| Figure 1E | | Figure 2I | |
| --- | --- | --- | --- |
| SLC22A3 |  | SLC7A11 |    |
| ACTB |  | GPX4 |  |
| Figure 5C | | Figure 5D | |
| MAOA |  | MAOA |  |
| MAOB |  | ACTB |  |
| ACTB |  |  |  |
| Figure 5H | | Figure 5G | |
| MAOA |    | MAOA |  |
| ACTB |  | ACTB |  |
| Figure 6B | | | |
| H3K4me |  | H3K4me2 |  |
| H3K4me3 |  | H3K4me3Q5 |  |
| ACTB |  |  |  |
| Figure 6K | | | |
| IB：WDR5 |  | IB：CHD1 |  |
| input |  | IB：TAF3 |  |
|  |  | input |  |
| Figure 7A | | | |
| IB:FOXO1 |  | Input |  |
| Figure 7D | | | |
| p-SIRT1 |  | SIRT1 |  |
| MAOA |  | FOXO1-ac |  |
| ACTB |  |  |  |
| FOXO1-ac |    | MAOA |  |
| SIRT1 |  | ACTB |  |
| FOXO1 |    |  |  |
| S1B | | S1G | |
| SLC22A3 |  | ACTB |  |
| S1C |  | S2E | |
| ACTB |  | H3K4me3 |    |
| SLC22A3 |  | SIRT1 |    |
|  |  | P-SIRT1 |    |
| S1H | | | |
| SLC22A3 |  | ACTB |  |
| S1E | | | |
| ACTB |  | SLC22A2 |  |
| SLC22A1 |  |  |  |
